# Mild-to-Moderate Kidney Dysfunction and Cardiovascular Disease: Observational and Mendelian Randomization Analyses

**DOI:** 10.1161/CIRCULATIONAHA.122.060700

**Published:** 2022-10-31

**Authors:** Liam Gaziano, Luanluan Sun, Matthew Arnold, Steven Bell, Kelly Cho, Stephen K. Kaptoge, Rebecca J. Song, Stephen Burgess, Daniel C. Posner, Katja Mosconi, Cassianne Robinson-Cohen, Amy M. Mason, Thomas R. Bolton, Ran Tao, Elias Allara, Petra Schubert, Lingyan Chen, James R. Staley, Natalie Staplin, Servet Altay, Pilar Amiano, Volker Arndt, Johan Ärnlöv, Elizabeth L.M. Barr, Cecilia Björkelund, Jolanda M.A. Boer, Hermann Brenner, Edoardo Casiglia, Paolo Chiodini, Jackie A. Cooper, Josef Coresh, Mary Cushman, Rachel Dankner, Karina W. Davidson, Renate T. de Jongh, Chiara Donfrancesco, Gunnar Engström, Heinz Freisling, Agustín Gómez de la Cámara, Vilmundur Gudnason, Graeme J. Hankey, Per-Olof Hansson, Alicia K. Heath, Ewout J. Hoorn, Hironori Imano, Simerjot K. Jassal, Rudolf Kaaks, Verena Katzke, Jussi Kauhanen, Stefan Kiechl, Wolfgang Koenig, Richard A. Kronmal, Cecilie Kyrø, Deborah A. Lawlor, Börje Ljungberg, Conor MacDonald, Giovanna Masala, Christa Meisinger, Olle Melander, Conchi Moreno Iribas, Toshiharu Ninomiya, Dorothea Nitsch, Børge G. Nordestgaard, Charlotte Onland-Moret, Luigi Palmieri, Dafina Petrova, Jose Ramón Quirós Garcia, Annika Rosengren, Carlotta Sacerdote, Masaru Sakurai, Carmen Santiuste, Matthias B. Schulze, Sabina Sieri, Johan Sundström, Valérie Tikhonoff, Anne Tjønneland, Tammy Tong, Rosario Tumino, Ioanna Tzoulaki, Yvonne T. van der Schouw, W.M. Monique Verschuren, Henry Völzke, Robert B. Wallace, S. Goya Wannamethee, Elisabete Weiderpass, Peter Willeit, Mark Woodward, Kazumasa Yamagishi, Raul Zamora-Ros, Elvis A. Akwo, Saiju Pyarajan, David R. Gagnon, Philip S. Tsao, Sumitra Muralidhar, Todd L. Edwards, Scott M. Damrauer, Jacob Joseph, Lisa Pennells, Peter W.F. Wilson, Seamus Harrison, Thomas A. Gaziano, Michael Inouye, Colin Baigent, Juan P. Casas, Claudia Langenberg, Nick Wareham, Elio Riboli, J.Michael Gaziano, John Danesh, Adriana M. Hung, Adam S. Butterworth, Angela M. Wood, Emanuele Di Angelantonio

**Affiliations:** Massachusetts Veterans Epidemiology Research and Information Center (MAVERIC), VA Boston Healthcare System, Boston, MA (L.G., K.C., R.J.S., D.C.P., P.S., J.J., J.P.C., J.M.G.).; BHF Cardiovascular Epidemiology Unit, Department of Public Health and Primary Care (L.G., L.S., S. Bell, S.K.K., S. Burgess, K.M., A.M.M., T.R.B., E.A., L.C., J.R.S., P.W., L. Pennells, S.H., M.I., J.D., A.S.B., A.M.W., E.D.A.); BHF Centre of Research Excellence, School of Clinical Medicine, Addenbrooke’s Hospital (A.M.M., S. Burgess, J.D., A.M.W., A.S.B., E.D.A.); Heart and Lung Research Institute, University of Cambridge, Cambridge UK (L.G., S. Bell, S.K.K., S. Burgess, K.M., A.M.M., E.A., L. Pennells, M.I., J.D., A.S.B., A.M.W., E.D.A.).; Medical Research Council Biostatistics Unit (A.M.M., S. Burgess), University of Cambridge, UK.; Stroke Research Group, Department of Clinical Neurosciences (S. Bell), University of Cambridge, UK.; NIHR Blood and Transplant Research Unit in Donor Health and Behaviour (S. Bell, T.R.B., E.A., J.D., A.S.B., A.M.W., E.D.A.), University of Cambridge, UK.; MRC Epidemiology Unit, School of Clinical Medicine (C.L., N.W.), University of Cambridge, UK.; Division of Aging (K.C., S.P., J.P.C. J.M.G.), Brigham and Women’s Hospital, Harvard Medical School, Boston, MA.; Division of Cardiovascular Medicine (J.J., T.A.G.), Brigham and Women’s Hospital, Harvard Medical School, Boston, MA.; Department of Epidemiology, Boston University School of Public Health, MA (R.J.S.).; Division of Nephrology, Department of Medicine (C.R.-C., E.A.A.), Vanderbilt University Medical Center, Nashville, TN.; Department of Biostatistics (R. Tao), Vanderbilt University Medical Center, Nashville, TN.; Medical Research Council Population Health Research Unit, Clinical Trial Service Unit and Epidemiological Studies Unit (N.S., C.B.), Nuffield Department of Population Health, University of Oxford, UK.; Cancer Epidemiology Unit (T.T.), Nuffield Department of Population Health, University of Oxford, UK.; Department of Cardiology, Trakya University School of Medicine, Edirne, Turkey (S.A.).; Ministry of Health of the Basque Government, Sub Directorate for Public Health and Addictions of Gipuzkoa, San Sebastián, Spain (P.A.).; Biodonostia Health Research Institute, Epidemiology of Chronic and Communicable Diseases Group, San Sebastián, Spain (P.A.).; Centro de Investigación Biomédica en Red de Epidemiología y Salud Pública (CIBERESP), Madrid, Spain (P.A., A.G.d.l.C., D.P., C. Santiuste).; Division of Clinical Epidemiology and Aging Research (V.A.), German Cancer Research Center (DKFZ), Heidelberg, Germany.; Department of Cancer Epidemiology (S.K.J., R.K., V.K.), German Cancer Research Center (DKFZ), Heidelberg, Germany.; Division of Family Medicine and Primary Care, Department of Neurobiology, Care Sciences and Society (NVS), Karolinska Institutet, Stockholm, Sweden (J.A., H.B.).; School of Health and Social Studies, Dalarna University, Falun, Sweden (J.A.).; Wellbeing & Preventable Chronic Diseases (WPCD) Division, Menzies School of Health Research, Charles Darwin University, Darwin, NT, Australia (E.L.M.B.).; Baker Heart and Diabetes Institute, Melbourne, VIC, Australia (E.L.M.B., M.I.).; Institute of Medicine, School of Public Health and Community Medicine (C.B.), Sahlgrenska Academy, University of Gothenburg, Sweden.; Institute of Medicine, Department of Molecular and Clinical Medicine (P.-O.H., A.R.), Sahlgrenska Academy, University of Gothenburg, Sweden.; National Institute for Public Health and the Environment (RIVM), Bilthoven, the Netherlands (J.M.A.B., W.M.M.V.).; Network Aging Research (NAR), Heidelberg University, Germany (H.B.).; Studium Patavinum (E.C.), University of Padua, Italy.; Department of Medicine (V.T.), University of Padua, Italy.; Dipartimento di Salute Mentale e Fisica e Medicina Preventiva, Università degli Studi della Campania ‘Luigi Vanvitelli’, Caserta, Italy (P.C.).; William Harvey Research Institute, NIHR Barts Biomedical Research Centre, Queen Mary University of London, UK (J.A.C.).; Johns Hopkins Bloomberg School of Public Health, Baltimore, MD (J.C.).; Larner College of Medicine, The University of Vermont, Burlington (M.C.).; The Gertner Institute for Epidemiology and Health Policy Research, Sheba Medical Center, Tel Hashomer, Israel (R.D.).; School of Public Health, Department of Epidemiology and Preventive Medicine, Tel Aviv University, Ramat Aviv, Tel Aviv, Israel (R.D.).; Feinstein Institutes for Medical Research, Northwell Health, Manhasset, New York, NY (R.D., K.W.D.).; Amsterdam University Medical Centers, VUMC, the Netherlands (R.T.d.J.).; Department of Cardiovascular, Endocrine-metabolic Diseases and Aging, Istituto Superiore di Sanità, Rome, Italy (C.D., L. Palmer).; Department of Clinical Sciences, Malmö, Lund University, Sweden (G.E., O.M.).; International Agency for Research on Cancer (IARC), World Health Organization, Lyon, France (H.F., E.W.).; 12 Octubre Hospital Research Institute, Madrid, Spain (A.G.d,l,C.).; Faculty of Medicine, University of Iceland, Reykjavik, Iceland and Icelandic Heart Association, Kopavogur, Iceland (V.G.).; Medical School Faculty of Health & Medical Sciences, The University of Western Australia, Perth, WA, Australia (G.J.H.).; Region Västra Götaland, Sahlgrenska University Hospital, Department of Medicine Geriatrics and Emergency Medicine/Östra, Gothenburg, Sweden (P.-O.H., A.R.).; School of Public Health (A.K.H., I.T., E.R.), Imperial College London, UK.; The George Institute for Global Health (M.W.), Imperial College London, UK.; Department of Internal Medicine, Division of Nephrology and Transplantation, Erasmus MC, University Medical Center Rotterdam, the Netherlands (E.J.H.).; Public Health, Department of Social Medicine, Osaka University Graduate School of Medicine, Suita, Japan (H.I.); University of Eastern Finland (UEF), Kuopio, Finland (J.K.).; Department of Neurology & Neurosurgery, Medical University of Innsbruck, Innsbruck, Austria (S.K.).; Clinical Epidemiology Team, Institute of Health Economics, Medical University of Innsbruck, Innsbruck, Austria (S.K., P.W.).; Institute of Epidemiology and Medical Biometry, University of Ulm, Germany (W.K.).; Deutsches Herzzentrum München, Technische Universität München, Germany (W.K.).; German Centre for Cardiovascular Research (DZHK), Partner Site Munich Heart Alliance (W.K.).; School of Public Health, University of Washington, Seattle (R.A.K.).; Danish Cancer Society Research Center, Copenhagen, Denmark (C.K., A.T.).; Medical Research Council Integrative Epidemiology Unit, University of Bristol, UK (D.A.L.).; Population Health Science, Bristol Medical School, UK (D.A.L.).; Department of Surgical and Perioperative sciences, Urology and Andrology, Umeå University, Sweden (B.L.).; University Paris-Saclay, UVSQ, Inserm, Villejuif, France (C. MacDonald).; Institute for Cancer Research, Prevention and Clinical Network (ISPRO), Florence, Italy (G.M.).; Helmholtz Zentrum München, Munich, Germany (C. Meisinger).; Navarra Public Health Institute, IdiSNA, Pamplona, Spain (C.M.I.).; Red de Investigación en Servicios de Salud en Enfermedades Crónicas (REDISSEC), Pamplona, Spain (C.M.I.).; Graduate School of Medical Sciences, Kyushu University, Fukuoka, Japan (T.N.).; London School of Hygiene & Tropical Medicine, UK (D.N.).; Herlev and Gentofte Hospital (B.G.N.), Copenhagen University Hospital, Copenhagen, Denmark.; Frederiksberg Hospital B.G.N.), Copenhagen University Hospital, Copenhagen, Denmark.; Department of Clinical Medicine, Faculty of Health and Medical Sciences (B.G.N.), University of Copenhagen, Denmark.; Department of Public Health (A.T.), University of Copenhagen, Denmark.; Julius Center for Health Sciences and Primary Care, University Medical Center Utrecht, Utrecht University, the Netherlands (C.O.-M., Y.T.v.d.S., W.M.M.V.).; Escuela Andaluza de Salud Pública (EASP), Granada, Spain (D.P.).; Instituto de Investigación Biosanitaria ibs.GRANADA, Granada, Spain (D.P.).; Consejería de Sanidad del Principado de Asturias Oviedo, Asturias, Spain (J.R.Q.G.).; Unit of Cancer Epidemiology, Città della Salute e della Scienza University-Hospital, Turin, Italy (C. Sacerdote).; Department of Social and Environmental Medicine, Kanazawa Medical University, Uchinada, Japan (M.S.).; Department of Epidemiology, Murcia Regional Health Council, IMIB-Arrixaca, Spain (C. Santiuste).; German Institute of Human Nutrition Potsdam-Rehbruecke, Nuthetal, Germany (M.B.S.).; German Center for Diabetes Research (DZD), Neuherberg, Germany (M.B.S.).; Institute of Nutritional Science, University of Potsdam, Germany (M.B.S.).; Fondazione IRCCS Istituto Nazionale dei Tumori di Milano, Milano, Italy (S.S.).; Department of Medical Sciences, Uppsala University, Sweden (J.S.).; Hyblean Association for Epidemiological Reserach AIRE - ONLUS, Ragusa, Italy (R.T.).; Universitätsmedizin Greifswald, Institut für Community Medicine, Abteilung SHIP/ Klinisch-Epidemiologische Forschung, Germany (H.V.).; College of Public Health, University of Iowa (R.B.W.).; University College London, UK (S.G.W.).; The George Institute for Global Health, Camperdown, NSW, Australia (M.W.).; Department of Public Health Medicine, Faculty of Medicine, and Health Services Research and Development Center, University of Tsukuba, Japan (K.Y.).; Unit of Nutrition and Cancer, Epidemiology Research Program, Catalan Institute of Oncology, Bellvitge Biomedical Research Institute (IDIBELL), L’Hospitalet de Llobregat (Barcelona), Spain (R.Z.-R.).; Center for Data and Computational Sciences, VA Boston Healthcare System, Boston, MA (S.P.).; Department of Biostatistics, Boston University School of Public Health, MA (D.R.G.).; VA Pal Alto Epidemiology Research and Information Center for Genomics, VA Palo Alto Health Care System, CA (P.S.T.).; Medicine (Cardiovascular Medicine), Stanford University of School of Medicine, CA (P.S.T.).; Office of Research and Development, Veterans Health Administration, Washington, DC (S.M.).; Department of Veterans Affairs, Tennessee Valley Health Care System, Vanderbilt University, Nashville (T.L.E.).; Medicine/Epidemiology, Vanderbilt Genetics Institute, Vanderbilt University Medical Center, Nashville, TN (T.L.E.).; Department of Surgery, Corporal Michael Crescenz VA Medical Center and Perelman School of Medicine, University of Pennsylvania, Philadelphia (S.M.D.).; Internal Medicine, VA Atlanta Healthcare System, Decatur, GA (P.W.F.W.).; Emory University School of Medicine (Cardiology), Emory University, Atlanta, GA (P.W.F.W.).; Center for Health Decision Science, Harvard T.H. Chan School of Public Health, Boston, MA (T.A.G.).; Health Data Research UK Cambridge, Wellcome Genome Campus and University of Cambridge, UK (M.I., J.D., A.S.B., A.M.W., E.D.A.); The Alan Turing Institute, London, UK (M.I.).; Computational Medicine, Berlin Institute of Health at Charité – Universitätsmedizin Berlin, Germany (C.L.).; Department of Human Genetics, Wellcome Sanger Institute, Hinxton, UK (J.D.).; Division of Nephrology & Hypertension, Department of Medicine, Tennessee Valley Health Care System and Vanderbilt University Medical Center, Nashville (A.M.H.).; Cambridge Centre for AI in Medicine, UK (A.M.W.).; Health Data Science Centre, Human Technopole, Milan, Italy (E.D.A.).

**Keywords:** cardiovascular diseases, coronary disease, kidney diseases, stroke

## Abstract

End-stage renal disease is associated with a high risk of cardiovascular events. It is unknown, however, whether mild-to-moderate kidney dysfunction is causally related to coronary heart disease (CHD) and stroke.

**Methods:**

Observational analyses were conducted using individual-level data from 4 population data sources (Emerging Risk Factors Collaboration, EPIC-CVD [European Prospective Investigation into Cancer and Nutrition–Cardiovascular Disease Study], Million Veteran Program, and UK Biobank), comprising 648 135 participants with no history of cardiovascular disease or diabetes at baseline, yielding 42 858 and 15 693 incident CHD and stroke events, respectively, during 6.8 million person-years of follow-up. Using a genetic risk score of 218 variants for estimated glomerular filtration rate (eGFR), we conducted Mendelian randomization analyses involving 413 718 participants (25 917 CHD and 8622 strokes) in EPIC-CVD, Million Veteran Program, and UK Biobank.

**Results:**

There were U-shaped observational associations of creatinine-based eGFR with CHD and stroke, with higher risk in participants with eGFR values <60 or >105 mL·min^–1^·1.73 m^–2^, compared with those with eGFR between 60 and 105 mL·min^–1^·1.73 m^–2^. Mendelian randomization analyses for CHD showed an association among participants with eGFR <60 mL·min^–1^·1.73 m^–2^, with a 14% (95% CI, 3%–27%) higher CHD risk per 5 mL·min^–1^·1.73 m^–2^ lower genetically predicted eGFR, but not for those with eGFR >105 mL·min^–1^·1.73 m^–2^. Results were not materially different after adjustment for factors associated with the eGFR genetic risk score, such as lipoprotein(a), triglycerides, hemoglobin A1c, and blood pressure. Mendelian randomization results for stroke were nonsignificant but broadly similar to those for CHD.

**Conclusions:**

In people without manifest cardiovascular disease or diabetes, mild-to-moderate kidney dysfunction is causally related to risk of CHD, highlighting the potential value of preventive approaches that preserve and modulate kidney function.

**Clinical Perspective**

**What Is New?**

- In people without manifest cardiovascular disease or diabetes, there is a nonlinear causal relationship between kidney function and coronary heart disease.
- Even mildly reduced kidney function is causally associated with higher risk of coronary heart disease with a possible risk threshold for eGFR value of ≈75 mL·min^–1^·1.73 m^–2^.
- The effect of reduced kidney function on coronary heart disease is independent of traditional cardiovascular risk factors.

**What Are the Clinical Implications?**

- Preventive approaches that can preserve and modulate kidney function can help prevent cardiovascular diseases.
- Given the nonlinear causal relationship, it may be a preferable strategy to identify individuals in the population with mild-to-moderate kidney dysfunction and target them for renoprotective interventions alongside routine strategies to reduce cardiovascular risk.

Chronic kidney disease (CKD), a major public health burden, affects >10% of the adult population globally.^1,2^ Kidney failure is associated with a high risk of cardiovascular disease (CVD) and all-cause mortality.^3–5^ Strong associations have also been reported between non–dialysis-dependent CKD and these outcomes in both people without manifest CVD and patients with ischemic CVD, heart failure, high blood pressure, or diabetes.^2,6,7^ These observations have led to guideline recommendations that patients with CKD should be regarded as being at very high risk of CVD.^8,9^

It is not known, however, whether mild-to-moderate kidney dysfunction is causally relevant to CVD or whether the increase in CVD risk associated with kidney dysfunction is related to changes in known risk factors, such as blood pressure and dyslipidemia, which seem to be a direct result of kidney dysfunction.^10–12^ An approach to help evaluate the causal relevance of kidney dysfunction to CVD is Mendelian randomization. Mendelian randomization uses genetic variants specifically related to a particular exposure to compare genetically defined population subgroups with different average levels of the exposure. The independent segregation of alleles at conception means that these genetically defined subgroups should not differ systematically with respect to confounding variables, creating a natural experiment analogous to a randomized trial. Therefore, compared with conventional observational analyses, Mendelian randomization analyses provide more reliable insights into causal relationships between risk factors and disease outcomes.^13,14^

Previous Mendelian randomization analyses that have assumed a linear dose-response relationship between kidney function and CVD have reported null associations.^14,15^ However, observational analyses have reported U-shaped associations of CVD risk with creatinine-based estimated glomerular filtration rate (eGFR), a measure of kidney function. Therefore, drawing on multiple large-scale population bioresources, we evaluated the causal relevance of eGFR to coronary heart disease (CHD) and stroke, using Mendelian randomization methods tailored to nonlinear relationships,^16–20^ which require concomitant information on eGFR, genetic determinants of eGFR, and first-ever CVD outcomes in the same individuals.

## Methods

The data, code, and study material that support the findings of this study are available from the corresponding author on reasonable request.

### Study Design and Study Overview

**Figure 1.**
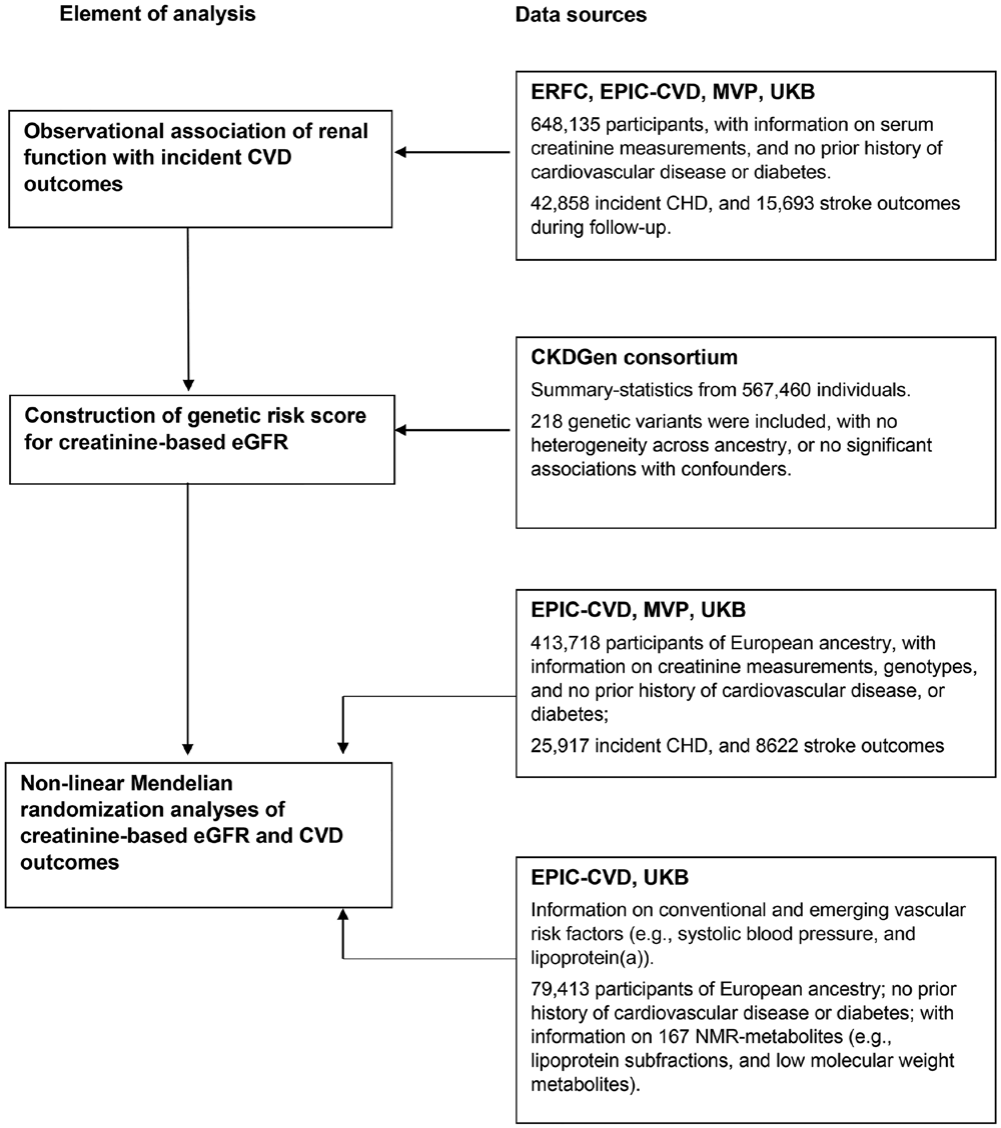
**Study design and overview.** CHD indicates coronary heart disease; CKDGen, CKD Genetics consortium; CVD, cardiovascular disease; eGFR, estimated glomerular filtration rate; EPIC-CVD, European Prospective Investigation into Cancer and Nutrition–Cardiovascular Disease; ERFC, Emerging Risk Factors Collaboration; MVP, Million Veteran Program; NMR, nuclear magnetic resonance; and UKB, UK Biobank.

This study involved interrelated components (Figure 1). First, we characterized observational associations between eGFR and incident CHD or stroke, using data from the Emerging Risk Factors Collaboration,^21^ EPIC-CVD (European Prospective Investigation into Cancer and Nutrition–Cardiovascular Disease Study),^22^ Million Veteran Program (MVP),^23^ UK Biobank (UKB),^24^ collectively involving 648 135 participants, who had serum creatinine measurements but no known CVD or diabetes at baseline. Second, we constructed a genetic risk score (GRS) for eGFR by computing a weighted sum of eGFR-associated index variants reported in a discovery genome-wide association study from the CKDGen consortium comprising 567 460 participants with European ancestry,^25^ none of whom were from MVP, EPIC-CVD, or UKB. Third, we used this GRS to conduct Mendelian randomization analyses in a total of 413 718 participants (ie, EPIC-CVD, MVP, UKB), with concomitant individual-level information on genetics, serum creatinine, and disease outcomes. Fourth, to assess the potential for interference by horizontal pleiotropy^26^ and explore potential mechanisms that could mediate associations between eGFR and CVD outcomes, we studied our GRS for eGFR in relation to several established and emerging risk factors for CVD.

### Data Sources

Information on each of the data sources used in the analysis is provided in the Expanded Methods in the Supplemental Material. In brief, Emerging Risk Factors Collaboration, a global consortium of population cohort studies with harmonized individual-participant data for multiple CVD risk factors, has included 47 studies with available information on serum creatinine and diabetes status at recruitment.^21^ EPIC-CVD, a case-cohort study embedded in the pan-European EPIC prospective study of >500 000 participants, has recorded data on serum creatinine and imputed genome-wide array data from 21 of its 23 recruitment centers.^22^ MVP, a prospective cohort study recruited from 63 Veterans Health Administration medical facilities throughout the United States, has recorded serum creatinine, and imputed genome-wide array data are available for a large subset of its participants.^23^ UKB, a prospective study of 22 recruitment centers across the United Kingdom, has cohort-wide information on serum creatinine and imputed genome-wide array data.^24^ Relevant ethical approval and participant consent were already obtained in all studies that contributed data to this work.

### Estimation of Kidney Function

Kidney function was estimated using creatinine-based eGFR, calculated using the Chronic Kidney Disease Epidemiology Collaboration equation.^27^ Creatinine concentration was multiplied by 0.95 for studies in which measurements were not standardized to isotope-dilution mass spectrometry.^25,28^ In a subset of participants with available data, kidney function was also defined using the Chronic Kidney Disease Epidemiology Collaboration cystatin C–based equation^29^ and albuminuria measured as spot urine albumin-to-creatinine ratio (Expanded Methods).

### Observational Analyses

Primary outcomes were incident CHD and stroke. Details of end-point definitions for each study are provided in Table S1. Participants in the contributing studies were eligible for inclusion in the present analysis if they met all of the following criteria: (1) aged 30 to 80 years at recruitment; (2) had recorded information on age, sex, circulating creatinine, and diabetes status; (3) had a creatinine-based eGFR of <300 mL·min^–1^·1.73 m^–2^; (4) did not have a known history of CVD or diabetes at baseline; (5) had complete information on the risk factors of smoking status, systolic blood pressure, total cholesterol, high-density lipoprotein cholesterol, and body mass index; and (6) had at least 1 year of follow-up data after recruitment.

Hazard ratios for associations of creatinine-based eGFR with incident CHD and stroke were calculated using Cox regression, stratified by sex and study center, and when appropriate, adjusted for traditional vascular risk factors (defined here as age, systolic blood pressure, smoking status, total cholesterol, high-density lipoprotein cholesterol, and body mass index) on a complete-case basis. To account for the EPIC-CVD case-cohort design, Cox models were adapted using Prentice weights.^30^ To avoid overfitting models, studies contributing <20 incident events to the analysis of a particular outcome were excluded from the analysis. Fractional polynomials were used to characterize nonlinear relationships of creatinine-based eGFR with risk of CHD and stroke, adjusted for age and CVD risk factors.^31^ Study-specific estimates for each outcome were pooled across studies using multivariable random-effects meta-analysis, using a reference point of 90 mL·min^–1^·1.73 m^–2^. When information on urinary biomarkers in UKB was available, participants were grouped into tenths on the basis of levels of urinary albumin-to-creatinine ratio to assess the shapes of associations between urinary biomarkers and CVD risk, using participants without albuminuria as the reference group.^32^

### GRS for Kidney Function

Using individual-participant data from EPIC-CVD, MVP, and UKB, we calculated a GRS^33^ weighted by the conditional effect estimated of the genetic variants associated (*P*<5×10^–8^) with creatinine-based eGFR in CKDGen,^25^ a global genetics consortium that has published genome-wide association study summary statistics for creatinine-based eGFR. Of the 262 variants associated with creatinine-based eGFR, 37 were excluded because of ancestry heterogeneity as reported in CKDGen,^25^ 4 were excluded because of associations (*P*<5×10^–8^) with vascular risk factors as reported in previous genome-wide association studies (ie, smoking status, alcohol consumption, and education attainment),^34^ and 3 were excluded because of missingness in at least 1 of the contributing studies, leaving 218 variants for the primary GRS for creatinine-based eGFR.

In sensitivity analysis, we constructed 2 restricted GRSs using 126 and 121 genetic variants that were likely to be relevant for kidney function on the basis of their associations with cystatin C–based eGFR^35^ and blood urine nitrogen,^25^ respectively. Sensitivity analysis was also conducted using a GRS that included all 262 transancestry eGFR-associated index variants. Furthermore, to evaluate traits that could mediate or confound (through horizontal pleiotropy) the associations between genetically predicted eGFR and outcomes, we tested associations of GRSs for eGFR with a range of cardiovascular risk factors in UKB and EPIC-CVD and with 167 metabolites measured using targeted high-throughput nuclear magnetic resonance metabolomics (Nightingale Health Ltd) in UKB.

### Mendelian Randomization Analyses

To account for the nonlinear relationship between eGFR and risk of CVD outcomes in observational analyses, we performed a stratified Mendelian randomization analysis using methods described previously.^16–20^ For each participant, we calculated the residual eGFR by subtracting the genetic contribution determined by the GRS from observed eGFR. Participants were grouped on the basis of their residual eGFR into 5-unit categories between 45 and <105 mL·min^–1^·1.73 m^–2^, plus <45 and ≥105 mL·min^–1^·1.73 m^–2^. By stratifying on residual eGFR, we compared individuals in the population who would have an eGFR in the same category if they had the same genotype and reduced the potential influence of collider bias. We then calculated Mendelian randomization estimates for each eGFR category using the ratio method with the GRS as an instrumental variable, adjusting for age, age-squared, sex, study center, and the first 10 principal components. Stratum-specific estimates were combined across studies using fixed-effect meta-analysis and plotted as a piecewise-linear function of eGFR, with pointwise confidence intervals calculated by resampling the stratum-specific estimates. Sensitivity analyses used non-parametric doubly-ranked stratification method. Detailed methods describing statistical analysis are in the Expanded Methods. Analyses used STATA 15.1 and R 3.6.1.

## Results

Among the 648 135 participants without history of CVD or diabetes at baseline, the mean age was 57 years, 57% were men, and 4.4% had creatinine-based eGFR <60 mL·min^–1^·1.73 m^–2^ (Table 1, Tables S2 and S3). During 6.8 million person-years of follow-up, there were 42 858 incident CHD outcomes and 15 693 strokes. Up to 413 718 participants of European ancestry from EPIC-CVD, MVP, and UKB contributed to the main genetic analyses (Figure 1). Distributions of serum creatinine concentration and creatinine-based eGFR were broadly similar across studies (Figures S1 and S2).

**Table 1.**
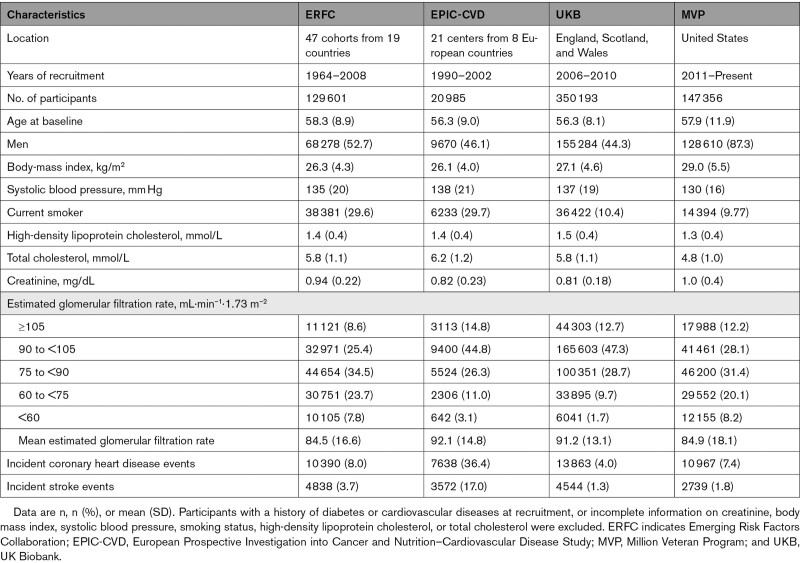
Study-Level and Participant-Level Characteristics of the Contributing Data Sources

### Observational Associations of eGFR With Cardiovascular Outcomes

**Figure 2.**
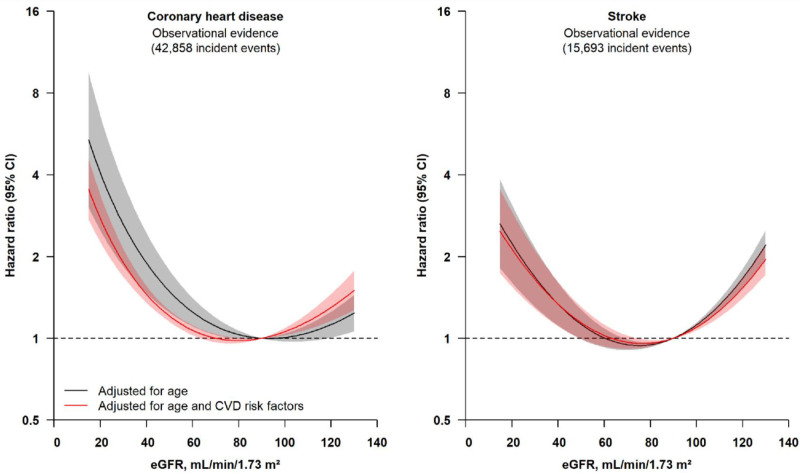
**Observational associations of eGFR levels with risk of coronary heart disease and stroke (n=648 135).** Participants with missing information on age and CVD risk factors (systolic blood pressure, total and high-density lipoprotein cholesterol, body mass index, and smoking status) were excluded from the analyses. Hazard ratios were estimated using Cox regression, adjusting for age and CVD risk factors (systolic blood pressure, total and high-density lipoprotein cholesterol, body mass index, and smoking status), and stratified by sex and study center. The reference point is 90 mL·min^–1^·1.73 m^–2^. Shaded regions indicate 95% CIs. CVD indicates cardiovascular disease; and eGFR, estimated glomerular filtration rate.

For both CHD and stroke, there were U-shaped associations of creatinine-based eGFR. Compared with participants with creatinine-based eGFR values between 60 and 105 mL·min^–1^·1.73 m^–2^, risks of both CHD and stroke were higher in people with eGFR <60 or >105 mL·min^–1^·1.73 m^–2^ (Figure 2, Figure S3). The shapes of these associations did not change substantially after adjustment for several traditional risk factors (Figure 2). Associations were similar in men and women, in clinically relevant subgroups (ie, smokers, people with obesity, or hypertension; Figure S4), in the different studies contributing to this analysis (Figure S5), and when participants with a history of diabetes or missing information on cardiovascular risk factors were included (Figures S6 and S7). Similar associations were also observed for ischemic stroke (Figure S3).

For the 338 044 participants in UKB with available data on serum cystatin C and urinary albumin-to-creatinine ratio, there were broadly similar associations of CHD or stroke with cystatin C–based eGFR as creatinine-based eGFR equations, but only when eGFR values were lower than ≈90 mL·min^–1^·1.73 m^–2^. However, there was no evidence of higher risk of CHD in participants with cystatin C–based eGFR values >105 mL·min^–1^·1.73 m^–2^ (Figure S8), in contrast with creatinine-based eGFR values >105 mL·min^–1^·1.73 m^–2^. Levels of urinary microalbumin and urinary albumin-to-creatinine ratio showed approximately linear associations with risk of CHD and stroke, which were somewhat attenuated after adjustment for traditional risk factors (Figure S9). Compared with participants with a creatinine-based eGFR of 75 to <90 mL·min^–1^·1.73 m^–2^ and without albuminuria, participants with albuminuria had higher risk of CHD and stroke (Figure S10).

### Mendelian Randomization of Genetically Predicted eGFR With Cardiovascular Outcomes

The GRS for eGFR (Table S4) explained 2.0% of variation in creatinine-based eGFR in EPIC-CVD, 2.2% in MVP, and 3.2% in UKB. A 1 SD increase in the GRS for eGFR was associated with 0.18 SD higher creatinine-based eGFR (Table S5, Figure S11). The GRS for eGFR was not associated with body mass index, diabetes, smoking status, or low-density lipoprotein cholesterol concentrations but showed modest associations with lipoprotein(a), triglycerides, blood pressure, and hemoglobin A1c measurement (Figure S11). Modest associations were also observed between the GRS for eGFR and triglyceride-related lipoprotein subclasses in a subset of participants with available data (Figure S12).

**Figure 3.**
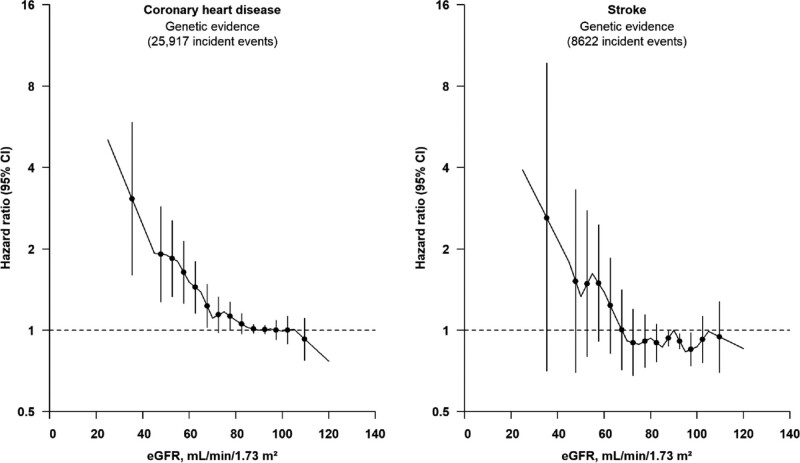
**Associations of genetically predicted eGFR with risk of coronary heart disease and stroke (n=413 718).** The reference point is 90 mL·min^–1^·1.73 m^–2^. Gradients at each point of the curve represent the localized average causal effect on coronary heart disease or stroke per 5 mL·min^–1^·1.73 m^–2^ change in genetically predicted eGFR. The vertical lines represent 95% CIs. Analyses were adjusted for age, age-squared, sex, study center, and the first 10 principal components of ancestry. eGFR indicates estimated glomerular filtration rate.

In nonlinear Mendelian randomization analysis, we observed a curvilinear relationship between genetically predicted eGFR and CHD (Figure 3). Among participants with eGFR <60 mL·min^–1^·1.73 m^–2^, each 5 mL·min^–1^·1.73 m^–2^ lower genetically predicted eGFR was associated with 14% (95% CI, 3%–27%) higher risk of CHD (Table 2). There was no clear evidence of association among participants with eGFR >75 mL·min^–1^·1.73 m^–2^ (Figure 3). Similar, but not statistically significant, associations were observed for stroke (Table 2, Figure 3). Overall, stratum-specific localized average causal estimates and nonlinear Mendelian randomization estimates were compatible across the studies contributing to this analysis (Table S6, Figure S13). Findings were supported in analyses using the non-parametric doubly-ranked stratification (Table S7, Figure S14). Similar associations were observed in analyses that further adjusted for systolic blood pressure, lipoprotein(a), hemoglobin A1c, and triglycerides (Figure S15), included participants with a history of diabetes at baseline (Figure S16), or used ischemic stroke as the stroke outcome (Figure S17). Results were also similar using GRS for cystatin C–based eGFR, blood urine nitrogen, or variants associated with creatinine-based eGFR regardless of ancestry heterogeneity (Figure S18).

**Table 2.**
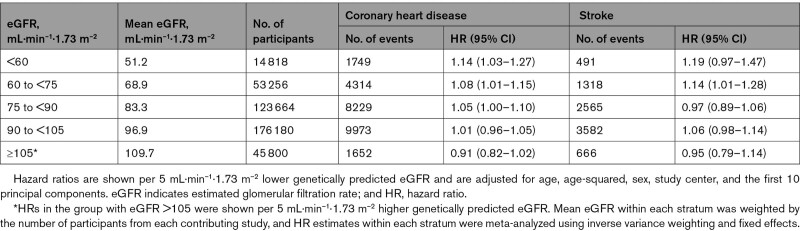
Mendelian Randomization Estimates per 5 mL·min^–1^·1.73 m^–2^ Lower Genetically Predicted eGFR With Risk of Coronary Heart Disease and Stroke

## Discussion

In analyses combining genetic, biomarker, and clinical data in ≈640 000 participants, our study has suggested that, in people without manifest CVD or diabetes, even mildly reduced kidney function is causally associated with a higher risk of CVD outcomes. Our results provide novel causal insights and highlight the wider potential value of preventive approaches that can preserve and modulate kidney function.

First, our study estimated a dose-response curve for genetically predicted eGFR and CHD, identifying an eGFR value of ≈75 mL·min^–1^·1.73 m^–2^ as a possible risk threshold. Therefore, the causal relationship of kidney function with CHD is nonlinear in shape, in contrast with those for blood pressure and low-density lipoprotein cholesterol, which each have approximately log-linear relationships with CHD risk across their range of values. In contrast with population-wide strategies to improve blood pressure and low-density lipoprotein cholesterol levels, this finding implies that it may be a preferable strategy to identify those in the population with mild-to-moderate kidney dysfunction and target them for renoprotective interventions alongside routine strategies to reduce cardiovascular risk. For example, the use of renoprotective interventions, such as renin angiotensin aldosterone system inhibitors^36^ and inhibitors of sodium-glucose cotransporter 2, might provide a potential means to do so.^37^ Our findings encourage additional evaluation of such agents in patients with CKD without manifest CVD or diabetes.^38,39^

Second, we found that our GRS for eGFR was modestly associated with several established and emerging CVD risk factors, including plasma concentration of proatherogenic lipids (eg, lipoprotein(a), triglycerides, and triglyceride-related lipoprotein subclasses), hemoglobin A1c values, and blood pressure, consistent with previous studies.^11,40^ However, adjustment for such factors did not materially alter the associations between eGFR and atherosclerotic CVD, indicating that they are unlikely to mediate or confound the associations between genetically predicted kidney dysfunction and CHD or stroke and limiting the likelihood that results are subject to influences of horizontal pleiotropy. These results suggest that the effect of reduced kidney function on CVD is independent of traditional cardiovascular risk factors and underscores the potential importance of direct preservation of renal function to prevent CVD, in addition to control of known risk factors.

Third, our data help to resolve controversies about the relevance to CHD of higher-than-average eGFR. In contrast with the observation that higher-than-average creatinine-based eGFR values are associated with higher CHD risk at >105 mL·min^–1^·1.73 m^–2^, we found that genetically predicted higher eGFR values were not associated with CHD risk in this same group. This discordance implies different pathophysiological meanings of creatinine-based eGFR values >105 mL·min^–1^·1.73 m^–2^ (which may represent a transient state of hyperfiltration before progression to poorer kidney function and CKD) and genetically predicted higher eGFR values (which represent a lifelong tendency toward exposure to better kidney function). This explanation is supported by our findings showing that the association between higher creatinine-based eGFR values and higher CHD risk was principally in participants who had albuminuria (and, therefore, preexisting kidney damage) at entry into the study.

Fourth, our results are broadly consistent with a causal relationship between eGFR and stroke. The lack of statistically significant findings in our Mendelian randomization analysis for stroke outcomes principally reflects the lower power of our study to evaluate a GRS with stroke compared with CHD. It may also be attributable to pathogenetic heterogeneity in stroke diagnoses (eg, cardioembolic, small vessel disease, and hemorrhagic subtypes may be less driven by atherosclerotic pathology than other ischemic stroke subtypes).^41,42^

Our study had major strengths, including a large sample size, access to individual-participant data, use of multiple genetic causal inference methods tailored to the evaluation of nonlinear disease associations, and an updated GRS that explains more variation in eGFR than previous analyses.^14^ However, there are also potential limitations. First, Mendelian randomization assumptions state that the only causal pathway from the genetic variants to the outcome is through eGFR. Although we assessed the potential for interference by horizontal pleiotropy, there is the possibility of residual confounding by unrecognized effects of genotypes on other risk factors and by adaptation during early life to compensate for genetically lower eGFR. Second, to reduce the scope for confounding by ancestry (population stratification), our analyses were limited to participants of European ancestries. This limitation means that our findings might not be applicable to other populations, and additional studies on this topic are needed, especially in non-European ancestry populations. Third, although serum creatinine is used routinely for estimating eGFR, true measurement of GFR requires the use of inulin, iohexol, or iothalamate. Assay of serum creatinine is liable to interference from other serum components (eg, bilirubin and glucose)^43,44^ and autoimmune activation^45^ and is sensitive to changes in individuals’ muscle mass (eg, sarcopenia). Assessment of cystatin C, an analyte that enables an alternative calculation of eGFR without the potential limitations of creatinine, was available only in a subset of the participants we studied. However, our genetic analyses restricted to genetic variants additionally associated with other biomarkers of kidney function showed results consistent with those for creatinine-based eGFR. Last, we used the 2009 Chronic Kidney Disease Epidemiology Collaboration equation to calculate eGFR. However, our analysis was limited to populations with European ancestry, in which the 2009 and 2021 Chronic Kidney Disease Epidemiology Collaboration equations provide similar estimates of eGFR.^46^

## Conclusions

In people without manifest CVD or diabetes, mild-to-moderate kidney dysfunction was causally related to cardiovascular outcomes, highlighting the potential cardiovascular benefit of preventive approaches that improve kidney function.

## Article Information

### Acknowledgments

The authors thank investigators and participants of the several studies that contributed data to the Emerging Risk Factors Collaboration. We thank all EPIC (European Prospective Investigation into Cancer) participants and staff for their contribution to the study, the laboratory teams at the Medical Research Council Epidemiology Unit for sample management and Cambridge Genomic Services for genotyping, S. Spackman for data management, and the team at the EPIC-CVD Coordinating Centre for study coordination and administration. The authors also thank the participants of the VA Million Veteran Program and its collaborators. Acknowledgment of VA Million Veteran Program leadership and staff contributions can be found in the Supplemental Material Note. This research has been conducted using the UK Biobank Resource under Application Number 31852.

### Sources of Funding

The Emerging Risk Factors Collaboration (ERFC) coordinating center was underpinned by program grants from the British Heart Foundation (BHF; SP/09/002; RG/13/13/30194; RG/18/13/33946), BHF Centre of Research Excellence (RE/18/1/34212), the UK Medical Research Council (MR/L003120/1), and the National Institute for Health and Care Research (NIHR) Cambridge Biomedical Research Centre (BRC-1215-20014), with project-specific support received from the UK NIHR, British United Provident Association UK Foundation, and an unrestricted educational grant from GlaxoSmithKline. This work was supported by Health Data Research UK, which is funded by the UK Medical Research Council, the Engineering and Physical Sciences Research Council, the Economic and Social Research Council, the Department of Health and Social Care (England), the Chief Scientist Office of the Scottish Government Health and Social Care Directorates, the Health and Social Care Research and Development Division (Welsh Government), the Public Health Agency (Northern Ireland), the BHF, and the Wellcome Trust. A variety of funding sources have supported recruitment, follow-up, and laboratory measurements in the studies contributing data to the ERFC, which are listed on the ERFC website (www.phpc.cam.ac.uk/ceu/erfc/list-of-studies). EPIC-CVD (European Prospective Investigation into Cancer and Nutrition–Cardiovascular Disease Study) was funded by the European Research Council (268834) and the European Commission Framework Programme 7 (HEALTH-F2-2012-279233). The coordination of EPIC is financially supported by International Agency for Research on Cancer (IARC) and also by the Department of Epidemiology and Biostatistics, School of Public Health, Imperial College London which has additional infrastructure support provided by the NIHR Imperial Biomedical Research Centre (BRC). The national cohorts are supported by: Danish Cancer Society (Denmark); Ligue Contre le Cancer, Institut Gustave Roussy, Mutuelle Générale de l’Education Nationale, Institut National de la Santé et de la Recherche Médicale (INSERM) (France); German Cancer Aid, German Cancer Research Center (DKFZ), German Institute of Human Nutrition PotsdamRehbruecke (DIfE), Federal Ministry of Education and Research (BMBF) (Germany); Associazione Italiana per la Ricerca sul Cancro-AIRC-Italy, Compagnia di SanPaolo and National Research Council (Italy); Dutch Ministry of Public Health, Welfare and Sports (VWS), Netherlands Cancer Registry (NKR), LK Research Funds, Dutch Prevention Funds, Dutch ZON (Zorg Onderzoek Nederland), World Cancer Research Fund (WCRF), Statistics Netherlands (The Netherlands); Health Research Fund (FIS) - Instituto de Salud Carlos III (ISCIII), Regional Governments of Andalucía, Asturias, Basque Country, Murcia and Navarra, and the Catalan Institute of Oncology - ICO (Spain); Swedish Cancer Society, Swedish Research Council and County Councils of Skåne and Västerbotten (Sweden); Cancer Research UK (14136 to EPIC-Norfolk; C8221/A29017 to EPIC-Oxford), Medical Research Council, United Kingdom (1000143 to EPIC-Norfolk; MR/M012190/1 to EPIC-Oxford). The establishment of the EPIC-InterAct subcohort (used in the EPIC-CVD study) and conduct of biochemical assays was supported by the EU Sixth Framework Programme (FP6) (grant LSHM_CT_2006_037197 to the InterAct project) and the Medical Research Council Epidemiology Unit (grants MC_UU_12015/1 and MC_UU_12015/5). This research is based on data from the Million Veteran Program, Office of Research and Development, and Veterans Health Administration and was supported by award I01-BX004821 (principal investigators, Drs Peter W.F. Wilson and Kelly Cho) and I01-BX003360 (principal investigators, Dr Adriana M. Hung). Dr Damrauer is supported by IK2-CX001780. Dr Hung is supported by CX001897. Dr Tsao is supported by BX003362-01 from VA Office of Research and Development. Dr Robinson-Cohen is supported by R01DK122075. Dr Sun was funded by a BHF Programme Grant (RG/18/13/33946). Dr Arnold was funded by a BHF Programme Grant (RG/18/13/33946). Dr Kaptoge is funded by a BHF Chair award (CH/12/2/29428). Dr Mason is funded by the EU/EFPIA Innovative Medicines Initiative Joint Undertaking BigData@Heart grant 116074. Dr Bolton was funded by the NIHR BTRU in Donor Health and Genomics (NIHR BTRU-2014-10024). Dr Allara is funded by a BHF Programme Grant (RG/18/13/33946). Prof Inouye is supported by the Munz Chair of Cardiovascular Prediction and Prevention and the NIHR Cambridge Biomedical Research Centre (BRC-1215-20014). Prof Inouye was also supported by the UK Economic and Social Research 878 Council (ES/T013192/1). Prof Danesh holds a British Heart Foundation Professorship and a NIHR Senior Investigator Award. Prof Wood is part of the BigData@Heart Consortium, funded by the Innovative Medicines Initiative-2 Joint Undertaking under grant agreement No 116074. Prof Wood was supported by the BHF-Turing Cardiovascular Data Science Award (BCDSA\100005). Prof Di Angelantonio holds a NIHR Senior Investigator Award.

### Disclosures

Where authors are identified as personnel of the International Agency for Research on Cancer/World Health Organization, the authors alone are responsible for the views expressed in this article, and they do not necessarily represent the decisions, policy, or views of the International Agency for Research on Cancer/World Health Organization. The views expressed are those of the author(s) and not necessarily those of the National Institute for Health Research or the Department of Health and Social Care. This publication does not represent the views of the Department of Veterans Affairs or the United States government. Dr Staley is now a full-time employee at UCB. Dr Sun is now an employee at Regeneron Pharmaceuticals. Dr Arnold is now an employee of AstraZeneca. Dr Danesh serves on scientific advisory boards for AstraZeneca, Novartis, and UK Biobank, and has received multiple grants from academic, charitable and industry sources outside of the submitted work. Adam Butterworth reports institutional grants from AstraZeneca, Bayer, Biogen, BioMarin, Bioverativ, Novartis, Regeneron and Sanofi.

### Supplemental Material

Expanded Methods

Tables S1–S7

Figures S1–S18

## Supplementary Material


