# Supplementary material for: Mild-to-Moderate Kidney Dysfunction and Cardiovascular Disease: Observational and Mendelian Randomization Analyses
Source: Circulation. 2022 Oct 31;146(20):1507–17. doi: 10.1161/CIRCULATIONAHA.122.060700 (PMC9662821; doi:10.1161/CIRCULATIONAHA.122.060700)
Supplement: Supplementary file 1 [file cir-146-1507-s001.pdf]

Emerging Risk Factors Collaboration Investigator list

ARIC: Anna Koettgen; **AUSDIAB**: Jonathan Shaw, Robert Atkins, Paul Zimmet; **BRHS**: Peter Whincup; **BRUN**: Peter Willeit, Johann Willeit, Christoph Leitner; **CASTEL**: Edoardo Casiglia, Valérie Tikhonoff; **COPEN**: Anne Tybjaerg-Hansen, Peter Schnohr, Shoaib Afzal; **DRECE**: David Lora Pablos, Cristina Martin Arriscado, Carmen Romero Ferreiro; **EPESEIOW**: Robert B Wallace; **ESTHER**: Hannah Stocker, Ben Schöttker, Bernd Holleczek; **GOH**: Angela Chetrit; **GOTO13**: Lennart Welin, Kurt Svärdsudd; **GOTO43**: Lennart Welin, Kurt Svärdsudd; **GOTOW**: Lauren Lissner, Dominique Hange, Kirsten Mehlig; **GRIPS**: Dorothea Nagel; **HIMS**: Paul E Norman, Osvaldo Almeida, Leon Flicker; **HISAYAMA**: Jun Hata, Takanori Honda, Yoshihiko Furuta; **IKNS**: Hiroyasu Iso, Akihiko Kitamura, Isao Muraki; **KIHD**: Jukka T Salonen, Tomi-Pekka Tuomainen; **LASA**: E M van Zutphen, N M van Schoor; **MATISS83**: Chiara Donfrancesco, Cinzia Lo Noce; **MATISS87**: Luigi Palmieri, Cinzia Lo Noce; **MESA**: Mary Cushman, Richard Kronmal; **MONICA_KORA3:** Wolfgang Koenig, Christa Meisinger; **MOSWEGOT**: Georg Lappas; **MPP**: Peter M Nilsson Olle Melander, Bo Hedblad; **MRCOLD**: Dorothea Nitsch; **NPHSII**: Jackie A Cooper; **NSHS**: Jonathan Shaffer Joseph Schwartz, Daichi Shimbo; **OSAKA**: Shinichi Sato Hiroyasu Iso, Mina Hayama-Terada; **RANCHO**: Simerjot Jassal; **REYK**: Thor Aspelund Bolli Thorsson, Gunnar Sigurdsson; **RS_I:** Layal Chaker, M. Kamran Ikram, Maryam Kavousi; **SHHEC**: Hugh Tunstall-Pedoe, Mark Woodward; **SHIP**: Henry Völzke; **TARFS**: Günay Can, Hüsniye Yüksel, Uğur Özkan; **TOYAMA**: Hideaki Nakagawa, Yuko Morikawa, Masao Ishizaki; **ULSAM**: Johan Ärnlöv; **WCWC**: Volker Arndt; **ZUTE**: Edith Feskens, Johanna M Geleijnse, Daan Kromhout.

Affiliations:

Anna Koettgen - Institute of Genetic Epidemiology, Department of Data Driven Medicine, Faculty of Medicine and Medical Center–University of Freiburg, Freiburg, Germany.

Jonathan Shaw – Baker Heart and Diabetes Institute, Melbourne, VIC, Australia and School of Public Health and Preventive Medicine, Monash University, Melbourne, VIC, Australia.

Robert Atkins – Monash University, Melbourne, Victoria, Australia.

Paul Zimmet – Department of Diabetes, Monash University, Melbourne, VIC, Australia.

Peter Whincup – Population Health Research Institute, St George's University of London, London, UK.

Peter Willeit – Clinical Epidemiology Team, Medical University of Innsbruck, Innsbruck, Austria.

Johann Willeit – Department of Neurology, Medical University of Innsbruck, Innsbruck, Austria.

Christoph Leitner – Institute of Health Care Engineering with European Testing Center of Medical Devices, Graz University of Technology, Graz, Austria and Institute of Sport Science, University of Graz, Graz, Austria.

Edoardo Casiglia – Department of Medicine, University of Padova, Padova, Italy.

Valérie Tikhonoff – Department of Medicine, University of Padova, Padova, Italy.

Anne Tybjaerg-Hansen – Department of Clinical Biochemistry, Rigshospitalet, Copenhagen University Hospital, Copenhagen, Denmark.

Peter Schnohr – Copenhagen City Heart Study Bispebjerg & Frederiksberg Hospital Copenhagen Denmark.

Shoaib Afzal – The Copenhagen General Population Study, Copenhagen University Hospital - Herlev and Gentofte, Copenhagen, Denmark; Faculty of Health and Medical Sciences, University of Copenhagen, Copenhagen, Denmark; Department of Clinical Biochemistry, Copenhagen University Hospital - Herlev and Gentofte, Copenhagen, Denmark.

David Lora Pablos – Scientific Support Unit, Instituto de Investigación Sanitaria Hospital Universitario 12 de Octubre (imas12), 28041 Madrid, Spain; Spanish Clinical Research Network (SCReN), 28040 Madrid, Spain; Consorcio de Investigación Biomédica en Red de epidemiología y salud pública (CIBEResp), 28029 Madrid, Spain; Faculty of Statistical Studies, Universidad Complutense de Madrid (UCM), 28040 Madrid, Spain.

Cristina Martin Arriscado – Clinical Research Department, Instituto de Investigación (imas12), Department of Surgery, Faculty of Medicine, Complutense University, Madrid, Spain.

Carmen Romero Ferreiro – Scientific Support Unit, Instituto de Investigación Sanitaria Hospital Universitario 12 de Octubre (imas12), 28041 Madrid, Spain; Spanish Clinical Research Network (SCReN), 28040 Madrid, Spain; Faculty of Health Sciences, Universidad Francisco de Vitoria, Pozuelo de Alarcón, 28223 Madrid, Spain.

Robert B Wallace – University of Iowa, Iowa City, Iowa, USA.

Hannah Stocker – Network Aging Research, Heidelberg University, Bergheimer Straße 20, 69115, Heidelberg, Germany; Division of Clinical Epidemiology and Aging Research, German Cancer Research Center, Im Neuenheimer Feld 581, 69120, Heidelberg, Germany; Medical Faculty, Heidelberg University, Im Neuenheimer Feld 672, 69120, Heidelberg, Germany.

Ben Schöttker – Division of Clinical Epidemiology and Aging Research, German Cancer Research Center (DKFZ), Im Neuenheimer Feld 280, 69120 Heidelberg, Germany; Network Aging Research, Heidelberg University, 69115 Heidelberg, Germany.

Bernd Holleczek – Division of Clinical Epidemiology Aging Research, German Cancer Research Centre (DKFZ), Heidelberg, Germany; Saarland Cancer Registry, Saarbrücken, Germany.

Angela Chetrit – Unit for Cardiovascular Epidemiology, the Gertner Institute for Epidemiology and Health Policy Research, Sheba Medical Center, Ramat Gan, Israel.

Lennart Welin – Department of Medicine, Lidköping Hospital, Lidköping, Sweden.

Kurt Svärdsudd – Department of Public Health and Caring Sciences, Uppsala University, Uppsala, Sweden.

Lauren Lissner – School of Public Health and Community Medicine, Institute of Medicine, Sahlgrenska Academy, University of Gothenburg, Göteborg, Sweden.

Dominique Hange – Primary Health Care/Department of Public Health and Community Medicine, Institute of Medicine, Sahlgrenska Academy, University of Gothenburg, Gothenburg, Sweden; Research, Education, Development & Innovation, Primary Health Care, Region Västra Götaland, Sweden.

Kirsten Mehlig – School of Public Health and Community Medicine, University of Gothenburg Institute of Medicine, Goteborg, Sweden.

Dorothea Nagel – Institute of Laboratory Medicine, Ludwig-Maximilians-University Munich, Munich, Germany.

Paul E Norman – Medical School, University of Western Australia, Perth, Western Australia, Australia (G.J.H., P.E.N.).

Osvaldo Almeida – UWA Medical School, The University of Western Australia, Perth, Western Australia, Australia.

Leon Flicker - Western Australian Centre for Health and Ageing, University of Western Australia, Perth, Western Australia, Australia.

Jun Hata – Department of Epidemiology and Public Health, Graduate School of Medical Sciences, Kyushu University, Fukuoka, Japan.

Takanori Honda - Department of Epidemiology and Public Health, Graduate School of Medical Sciences Kyushu University Fukuoka Japan.

Yoshihiko Furuta - Department of Epidemiology and Public Health, Graduate School of Medical Sciences Kyushu University Fukuoka Japan; Department of Medicine and Clinical Science, Graduate School of Medical Sciences Kyushu University Fukuoka Japan.

Hiroyasu Iso - Public Health, Department of Social and Environmental Medicine, Osaka University Graduate School of Medicine, Suita, Japan.

Akihiko Kitamura - Osaka Center for Cancer and Cardiovascular Disease Prevention, Osaka, Japan; Yao City Public Health Center, Yao, Japan.

Isao Muraki - Public Health, Department of Social Medicine, Osaka University Graduate School of Medicine.

Jukka T Salonen - University of Helsinki, the Faculty of Medicine, Department of Public Health, Helsinki, Finland; Metabolic Analytical Services Oy, Helsinki, Finland.

Tomi-Pekka Tuomainen - Institute of Public Health and Clinical Nutrition, University of Eastern Finland, Kuopio, Finland.

E M van Zutphen – Amsterdam UMC, Department of Epidemiology and Data Science, Amsterdam.

N M van Schoor – Amsterdam UMC, Department of Epidemiology and Data Science, Amsterdam.

Chiara Donfrancesco - Istituto Superiore di Sanità, Rome, Italy.

Cinzia Lo Noce - Department of Cardiovascular, Endocrine-Metabolic Diseases and Aging, Istituto Superiore di Sanità, 00161 Rome, Italy.

Luigi Palmieri - Department of Cardiovascular, Endocrine-Metabolic Diseases and Aging, Istituto Superiore di Sanità, 00161 Rome, Italy.

Mary Cushman - Department of Medicine, Larner College of Medicine at the University of Vermont, Burlington, VT, USA.

Richard Kronmal - Department of Biostatistics University of Washington School of Public Health Seattle Washington USA.

Wolfgang Koenig - Deutsches Herzzentrum München, Technische Universität München, Munich, Germany; German Center for Cardiovascular Disease Research (DZHK), partner site Munich Heart Alliance, Munich, Germany; Institute of Epidemiology and Medical Biometry, University of Ulm, Ulm, Germany.

Christa Meisinger - Chair of Epidemiology, University of Augsburg, at University Hospital Augsburg, Augsburg, Germany.

Georg Lappas - Department of Molecular and Clinical Medicine, Sahlgrenska Academy, University of Gothenburg and Sahlgrenska University Hospital, Sweden.

Peter M Nilsson - Department of Clinical Sciences, Skane University Hospital, Lund University, Malmö, Sweden.

Olle Melander - Department of Clinical Sciences Malmö, Lund University, Skåne University Hospital, Malmö, Sweden.

Bo Hedblad – Department of Clinical Sciences in Malmö, CRC, Lund University and Skåne University Hospital, Malmö, Sweden.

Dorothea Nitsch - Department of Non-Communicable Disease Epidemiology, London School of Hygiene and Tropical Medicine, London, UK.

Jackie A Cooper - NIHR Biomedical Research Centre at Barts, William Harvey Research Institute, Queen Mary University of London, London E1 4NS, UK.

Jonathan Shaffer - Department of Psychology University of Colorado Denver Denver CO.

Joseph Schwartz - Mount Sinai Health System, Tenafly, New Jersey, United States.

Daichi Shimbo - Department of Medicine, Columbia University Irving Medical Center.

Shinichi Sato - Graduate School of Life Sciences, Tohoku University, 2-1-1 Katahira, Aoba-ku, Sendai 980-8577, Japan; Frontier Research Institute for Interdisciplinary Sciences, Tohoku University, 2-1-1 Katahira, Aoba-ku, Sendai 980-8577, Japan.

Mina Hayama-Terada - Osaka Center for Cancer and Cardiovascular Disease Prevention, Osaka, Japan; Yao City Public Health Center, Yao, Japan.

Simerjot Jassal - University of California San Diego, La Jolla, CA, USA; San Diego VA Health Care System, San Diego, CA, USA.

Thor Aspelund - Faculty of Medicine, School of Health Sciences, University of Iceland, Reykjavik, Iceland.

Bolli Thorsson – Faculty of Medicine, School of Health Sciences, University of Iceland, The Icelandic Heart Association.

Gunnar Sigurdsson - Icelandic Heart Association Research Institute, Kopavogur, Iceland.

Layal Chaker - Dept. of Internal Medicine, Erasmus University Medical Center, Rotterdam, the Netherlands.

M. Kamran Ikram - Department of Epidemiology, Erasmus University Medical Center Rotterdam, PO Box 2040, 3000 CA, Rotterdam, The Netherlands; Department of Neurology, Erasmus University

Medical Center Rotterdam, Rotterdam, The Netherlands.

Maryam Kavousi - Department of Epidemiology, Erasmus MC University Medical Center Rotterdam, Rotterdam, The Netherlands.

Hugh Tunstall-Pedoe - Cardiovascular Epidemiology Unit, Institute of Cardiovascular Research, University of Dundee, Dundee, United Kingdom.

Mark Woodward - The George Institute for Global Health, School of Public Health, Imperial College, London, United Kingdom; School of Public Health, Imperial College, London, United Kingdom.

Henry Völzke - Institute for Community Medicine, University Medicine Greifswald, Greifswald, Germany.

Günay Can - Department of Public Health, Division of Endocrinology and Metabolism, Istanbul University-Cerrahpasa, Cerrahpasa Medical Faculty, Istanbul, Turkey.

Hüsniye Yüksel - Ataşehir Florence Nightingale Hospital, Istanbul, Turkey.

Uğur Özkan - Trakya University, Cardiology Department, Edirne, Turkey.

Hideaki Nakagawa - Department of Social and Environmental Medicine, Kanazawa Medical University, Uchinada 920-0293, Japan.

Yuko Morikawa - Department of Radiological Technology, Tsuchiya General Hospital, Nakajima-cho 3-30, Naka-ku, Hiroshima 730-8655, Japan.

Masao Ishizaki - Department of Hygiene, Kanazawa Medical University, Uchinada, Japan.

Johan Ärnlöv - Division of Family Medicine and Primary Care, Department of Neurobiology, Care Science and Society, Karolinska Institute, Huddinge, Sweden; School of Health and Social Studies, Dalarna University, Falun, Sweden.

Volker Arndt - Unit of Cancer Survivorship, Division of Clinical Epidemiology and Aging Research, German Cancer Research Center (DKFZ), PO Box 101949, 69009, Heidelberg, Germany.

Edith Feskens - Division of Human Nutrition and Health, Wageningen University, Wageningen, Netherlands.

Johanna M Geleijnse - Division of Human Nutrition and Health, Wageningen University and Research, Stippeneng 4, P.O. Box 176700, AA Wageningen, 6708 WE Wageningen, The Netherlands.

Daan Kromhout - Division of Human Nutrition and Health Wageningen University Wageningen the Netherlands; Department of Epidemiology University Medical Center Groningen Groningen the Netherlands.
