# Supplementary material for: Mild-to-Moderate Kidney Dysfunction and Cardiovascular Disease: Observational and Mendelian Randomization Analyses
Source: Circulation. 2022 Oct 31;146(20):1507–17. doi: 10.1161/CIRCULATIONAHA.122.060700 (PMC9662821; doi:10.1161/CIRCULATIONAHA.122.060700)
Supplement: Supplementary file 2 [file cir-146-1507-s002.pdf]

Mild-to-moderate kidney dysfunction and cardiovascular disease: observational and Mendelian randomization analyses

Running Title: Gaziano et al.; *Kidney dysfunction and CVDs*

Supplementary Materials

Contents | Page

Contents	Page
Expanded Methods	2
Supplementary Tables	7
Supplementary Figures	17
Supplementary Acknowledgements	34

EXPANDED METHODS

I. Description of contributing studies or consortium

Emerging Risk Factor Collaboration

Emerging Risk Factor Collaboration (ERFC) is a consortium of 112 prospective studies, involving a total of 1.2 million participants, that provided individual-level data.[21] These studies were approximately population-based (i.e., did not select participants on the basis of having previous cardiovascular disease); recorded cause-specific mortality or vascular morbidity using accepted criteria; and had accrued more than 1 year of follow-up. Coronary heart disease (CHD) and stroke were defined in each contributing study. Sixty-two studies used standard definitions of myocardial infarction (MI) based on World Health Organization criteria. Fifty-six studies reported diagnosis of strokes on the basis of typical clinical features and characteristic changes on brain imaging, and all attempted to provide attribution of stroke pathological types. In registering fatal outcomes, all contributing studies used coding from the *International Classification of Diseases* (ICD) to at least 3 digits and ascertainment was based on death certificates. Data on serum creatinine measurements were available in 48 studies, and were harmonized at the ERFC coordinating centers in consensus with the individual study collaborators. Genetic information was not available for the current analysis on any of the contributing studies, and therefore ERFC was not included in the Mendelian randomization (MR) analysis.

Million Veteran Program

Million Veteran Program (MVP) is a prospective biobank with ongoing recruitment from 63 Veterans Health Administration (VA) medical facilities that started in 2011.[23] Participant questionnaires and linkage to Electronic Health Records (EHR) from the VA healthcare system, national death index (NDI), and Centers for Medicare and Medicaid Services (CMS) were used to define baseline exposures and case status.[47-49] CHD was defined as ICD-9 410-414, or ICD-10 I20-I25 and stroke was defined as ICD-9 430-431 or 433-434, or ICD-10 I60-I61 or I63, I69. Creatinine was extracted from EHR as the value closest but prior to enrolment up to a year. Anyone with CHD or stroke codes prior to enrolment were excluded, along with amputees and individuals on HIV medications.[23] Genotyping was performed using an array similar to the UK Biobank Affymetrix Axiom array but with modifications tailored to the veteran population.[47] Genotypes were imputed with Minimac3,[48] using the 1000 Genomes Project reference panel (phase 3, version 5),[49] after phasing by EAGLE v2 software.[50] Ancestry was determined with HARE (harmonized ancestry and race/ethnicity) software, which allocates individuals into ancestry groups from a combination of self-identified race/ethnicity and genetic information.[51] The VA central institutional review board and site-specific Research and Development committees approved the Million Veteran Program study.

UK Biobank

Details of the design, methods, and participants of UK Biobank (UKB) have been described previously.[52] Briefly, participants aged 40 to 75 years identified through primary care lists were recruited across 22 assessment centers throughout the UK between 2006 and 2010. At recruitment, information was collected via a standardized questionnaire and selected physical measurements. Data were subsequently linked to Hospital Episode Statistics (HES), as well as national death and cancer registries. HES uses ICD–9th and 10th Revisions to record diagnosis information, and *Office of Population, Censuses and Surveys: Classification of Interventions and Procedures*, version 4 (OPCS-4) to code operative procedures. Death registries include deaths in the UK, with both primary and contributory causes of death coded in ICD-10. CHD was defined as ICD-10 I20-I25 and stroke was defined as ICD-10 I60-I61 or I63, I64, I69. Genotyping was undertaken using a custom-built genome-wide array of ~826,000 markers.[24] Imputation to ~96 million markers was subsequently carried out using the Haplotype Reference Consortium and UK10K/1000Genomes reference panels.[24] Clinical biochemistry markers, including blood creatinine, total cholesterol, HDL-cholesterol, urinary albumin, and urinary creatinine, were measured in bio-samples collected at baseline. Full details of the biochemistry sampling, handling and quality control protocol, and assay method has been described previously.[53]

EPIC-CVD

EPIC-CVD is a case-cohort embedded in the European Prospective Investigation into Cancer and Nutrition (EPIC), to advance understanding about the separate and combined influence of lifestyle, biochemical, and genetic factors in the development of cardiovascular disease.[54] Briefly, between 1992 and 2000, 519,978 participants were recruited to the EPIC prospective study across 23 centers in ten European countries, via population-based registers, blood donors, screening clinics. CHD and stroke cases were ascertained at each recruiting center through death registries, hospital discharge codes, self-reported by questionnaires or through active follow-up by correspondence with relatives for fatal events. Within each of the contributing EPIC centers, information has been collected and centrally harmonized at the EPIC-CVD Coordinating Centre on: i) a random sample of the original center-specific cohort (i.e., the “sub-cohort”), and ii) all incident CHD and stroke cases. Participants were genotyped using either the Illumina 660W-Quad BeadChip at the Wellcome Trust Sanger Institute or the Illumina HumanCoreExome BeadChip at Cambridge Genomic Services. Samples from each array were then imputed separately to the Haplotype Reference Consortium panel,[55] using IMPUTE2 software.[56]

II. ERFC study acronyms

Abbreviation	Full Name
ARIC	Atherosclerosis Risk in Communities Study
AUSDIAB	Australian Diabetes, Obesity and Lifestyle study’
BRHS	British Regional Heart Study
BRUN	Bruneck Study
BWHHS	British Women's Heart and Health Study
CASTEL	Cardiovascular Study in the Elderly
CHS1	Cardiovascular Health Study - 1
CHS2	Cardiovascular Health Study - 2
COPEN	Copenhagen City Heart Study
DRECE	Diet and Risk of Cardiovascular Disease in Spain
EPESEBOS	Established Populations for the Epidemiologic Study of the Elderly Studies, East Boston
EPESEIOW	Established Populations for the Epidemiologic Study of the Elderly Studies, Iowa
EPESENCA	Established Populations for the Epidemiologic Study of the Elderly Studies, North Carolina
EPESENHA	Established Populations for the Epidemiologic Study of the Elderly Studies, New Haven
ESTHER	Epidemiologische Studie zu Chancen der Verhutung und optimierten Therapie chronischer Erkrankungen in der alteren Bevolkerung
GOH	The Glucose Intolerance, Obesity and Hypertension Study
GOTO13	Goteborg Study 1913
GOTO43	Goteborg Study 1943
GOTOW	Population Study of Women in Göteborg, Sweden
GRIPS	Göttingen Risk Incidence and Prevalence Study
HIMS	Health in Men Study
HISAYAMA	Hisayama Study
IKNS	Ikawa, Kyowa, and Noichi Study
KIHD	Kuopio Ischaemic Heart Disease Study
LASA	Longitudinal Aging Study Amsterdam
MATISS83	Progetto CUORE
MATISS87	Progetto CUORE
MESA	Multi-Ethnic Study of Atherosclerosis
MONICA_KORA3	MONICA/KORA Augsburg Survey S3
MOSWEGOT	MONICA Göteborg Study
MPP	Malmö Preventive Project
MRCOLD	MRC Study of Older People
NHANESI	National Health and Nutrition Examination Survey I
NHANESIII	National Health and Nutrition Examination Survey III
NPHSII	Northwick Park Heart Study II
NSHS	Nova Scotia Health Survey
OSAKA	Osaka Study
RANCHO	Rancho Bernardo Study
REYK	Reykjavik Study
RS_I	The Rotterdam Study I
SHHEC	Scottish Heart Health Extended Cohort
SHIP	Study of Health in Pomerani
TARFS	Turkish Adult Risk Factor Study
TOYAMA	Toyama Study
ULSAM	Uppsala Longitudinal Study of Adult Men
WCWC	Wuertemberg Construction Workers Cohort
ZUTE	Zutphen Elderly Study

III. Estimators of glomerular filtration rates

We used eGFR from serum creatinine predicted from CKD-EPI formula in the primary analyses, and where available, we compared results with eGFR estimated using other formulae.

Estimating equations using serum creatinine in mg/dL (to convert serum creatinine in mmol/L to mg/dL, divide by 88.4):

1. Chronic Kidney Disease Epidemiology Collaboration (CKD-EPI)[57]:

$$\text{eGFR} = 141 \times \left\{\min\left(\frac{\text{creatinine}}{k}, 1\right)\right\}^{\alpha} \times \left\{\max\left(\frac{\text{creatinine}}{k}, 1\right)\right\}^{-1.209} \times 0.993^{\text{age}} \times [1.018 \text{ if female}] \times [1.159 \text{ if black}]$$

where $k = 0.7$ and $\alpha = -0.329$ for females, and $k = 0.9$ and $\alpha = -0.411$ for males.

Estimating equations using serum cystatin C in mg/L[27]:

1. CKD-EPI

$$\text{eGFR} = 130 \times \left(\frac{\text{cystatin C}}{0.8}\right)^{\alpha} \times 0.996^{\text{age}} \times [0.932 \text{ if female}]$$

where $\alpha = -0.499$ if cystatin C ≤ 0.8, and $\alpha = -1.328$ otherwise.

Estimating equations using serum creatinine and serum cystatin C:

1. CKD-EPI

$$\text{eGFR} = 135 \times \left\{\min\left(\frac{\text{creatinine}}{k}, 1\right)\right\}^{\alpha} \times \left\{\max\left(\frac{\text{creatinine}}{k}, 1\right)\right\}^{-0.601} \times \left(\frac{\text{cystatin C}}{0.8}\right)^{\beta} \times 0.995^{\text{age}} \times [0.969 \text{ if female}] \times [1.08 \text{ if black}]$$

where $k = 0.7$ and $\alpha = -0.248$ for females, and $k = 0.9$ and $\alpha = -0.207$ for males, and $\beta = -0.375$ if cystatin C ≤ 0.8, and $\beta = -0.711$ otherwise

IV. Non-linear Mendelian randomization analysis

Mendelian randomization (MR) methods typically assume that the exposure-outcome relationship is linear when estimating a causal association. However, large-scale prospective epidemiological studies among different populations, and the present study performed among participants without prior history of cardiovascular disease or diabetes, have demonstrated that there is a reversed J-shaped association between creatinine-based eGFR and risk of CHD and stroke. To account for the non-linear relationship when investigating the causal relevance of kidney function for risk of CHD, and stroke, we applied a tailored novel method, i.e., the non-linear MR approach.[16-17,58]

Genetically-predicted eGFR and cardiovascular outcomes

Within each study (i.e., EPIC-CVD, MVP, and UKB), we calculated the residual variation in creatinine-based eGFR (henceforth, termed as *IV-free* eGFR) by subtracting the genetically-determined eGFR from the creatinine-based eGFR. The IV-free eGFR can be interpreted as the expected value of a participant's eGFR if their GRS was 0. Based on the IV-free eGFR, all study participants were then stratified into 5-unit groups between 45-105 mL/min/1.73 m^2, plus <45 and ≥105 mL/min/1.73 m^2. Within stratum, linear MR estimates were calculated using the ratio method. These stratum-specific MR estimates are localized average causal effect (LACE) estimates and were pooled across studies using fixed-effects meta-analysis within each stratum. The stratum-specific MR estimates were plotted as a piecewise-linear function of eGFR, where the slope in each piece is the LACE estimate in that stratum. The estimated risk was plotted against the mean eGFR in each stratum, relative to the risk at 90 mL/min/1.73 m^2. Point-wise confidence intervals were calculated by re-sampling the MR estimates and re-calculating the full piecewise-linear shape. Interpretation of graphical representations of these non-linear genetic associations must focus on the slope in the neighborhood of an eGFR value of interest, rather than comparisons of absolute risk made across the range of the eGFR distribution.

Genetically-predicted eGFR and other vascular risk factors

To assess the specificity of the GRSs for eGFR, we tested associations of GRSs with a range of vascular risk factors (e.g., systolic blood pressure, LDL-cholesterol, and diabetes status) in UKB and EPIC-CVD studies, and with 167 metabolites measured using targeted high-throughput NMR metabolomics (Nightingale Health Ltd) in UKB. The analyses were conducted among participants with no prior history of vascular diseases or diabetes, and not on lipid-lowering treatments. NMR-measured metabolites were standardized for comparison via rank-based inverse normal transformation. Linear regression was used to relate GRSs to continuous traits, and logistic regression was used for binary outcomes, adjusted for age, age-squared, sex, study center, and the first ten principal components.

Sensitivity analyses using doubly-ranked method

An assumption made in the primary non-linear Mendelian randomization method described above (hereafter referred to as the "residual method") is that the effect of the genetic instrument on the exposure is constant across the distribution of the exposure. That is, the magnitude of the genetic effect on eGFR is the same for individuals with different values of eGFR. A recently-developed method for non-linear Mendelian randomization, the "doubly-ranked method", is able to relax this assumption[59]. However, the doubly-ranked method has some practical limitations in the context of this investigation. In particular, strata created by the residual method can be defined with respect to clinically-relevant eGFR values, whereas strata created by the doubly-ranked method cannot. This means that estimates from the doubly-ranked method cannot easily be combined across datasets. However, estimates from the residual method are subject to potential bias when the constant genetic effect assumption is violated, although the extent of bias is unclear. We implemented both the doubly-ranked and residual methods to assess the robustness of findings to this assumption. For both methods, we divided the population into deciles and calculated Mendelian randomization estimates in each decile for the outcome of CHD.

Results are displayed in Table S6a and Figure S13a. We observe evidence that the constant genetic effect assumption is violated, although the violation is stronger at the top end of the eGFR distribution compared with the bottom end. The estimates from the residual method indicate that there is evidence for a causal effect of eGFR on CHD risk from the doubly-ranked method in the lowest three deciles for MVP, and some evidence in the lowest decile for UK Biobank. In both cases, these are the only decile groups with a mean eGFR below 75 mL/min/1.73 m^2. There is also some evidence for a causal effect of eGFR in the highest decile groups for both methods. Overall, results are similar from the residual and doubly-ranked methods, suggesting that bias due to violation of the constant genetic effect assumption is not substantial in this case.

Table S1: Definitions of coronary heart diseases and stroke

Outcome (includes both fatal and non-fatal)	ICD-10 codes
**All cardiovascular**	I20-I25, I60-I64, I69
**Coronary heart disease**	I20-I25
**All stroke**	I60-I64, I69
Ischemic stroke	I63, I69.3
Intracerebral hemorrhage	I61, I69.1
Subarachnoid hemorrhage	I60, I69.0
Unclassified stroke †	I64, I69.4

† Unclassified stroke refers to ICD codes I64 (ICD-10), 436 (ICD-9) or earlier ICD equivalents, or strokes no specified as ischemic or hemorrhagic stroke in study specific codes. Corresponding ICD-6, 7 or 8 codes are used for ERFC studies that recorded outcomes using earlier ICD versions.

Table S2: Number of participants by eGFR category and contributing study

Cohort	Total	creatinine-based eGFR categories, mL/min/1.73 m^2						
		<45	45-<60	60-<75	75-<90	90-<105	105-<120	>120
ARIC	11,421	64	1,271	5,134	3,719	1,054	164	15
AUSDIAB	8,307	35	338	1,807	3,502	2,176	447	2
BRHS	6,406	15	180	1,529	2,785	1,646	249	2
BRUN	756	4	20	98	265	297	72	-
BWHHS	2,503	17	306	1,137	885	157	1	-
CASTEL	1,793	54	224	464	746	292	10	3
CHS1	2,936	127	613	1,048	896	246	6	-
CHS2	312	9	28	91	89	75	19	1
COPEN	6,569	94	819	2,277	2,192	1,004	179	4
DRECE	2,058	4	27	312	762	663	265	25
EPESEBOS	437	55	179	138	61	4	-	-
EPESEIOW	708	112	304	193	94	5	-	-
EPESENCA	609	105	260	197	42	5	-	-
EPESENHA	341	46	134	108	43	8	2	-
ESTHER	3,995	134	372	646	984	1,425	349	85
GOH	867	12	42	200	248	257	106	2
GOTO43	723	-	1	56	303	350	12	1
GRIPS	5,645	14	184	1,160	2,720	1,266	297	4
HIMS	1,823	47	152	480	984	158	2	-
HISAYAMA	2,236	12	173	825	858	334	34	-
IKNS	4,204	12	68	415	1,139	1,847	708	15
KIHD	1,792	5	13	209	627	796	137	5
LASA	124	3	26	66	27	2	-	-
MATISS83	2,403	10	70	297	576	899	479	72
MATISS87	1,876	17	46	268	500	669	341	35
MESA	5,696	56	319	1,204	2,021	1,672	381	43
MONICA_KORA3	3,902	16	39	154	457	1,387	1,441	408
MOSWEGOT	309	5	93	137	63	11	-	-
NHANESIII	8,946	156	853	2,322	3,079	1,896	544	96
NSHS	913	6	16	75	168	374	230	44
OSAKA	3,476	9	39	477	1,296	1,244	394	17
RANCHO	1,406	71	424	546	286	76	3	-
REYK	11,808	38	388	2,640	4,650	3,551	529	12
RS_I	3,371	18	213	915	1,434	777	13	1
SHHEC	9,643	23	282	2,402	4,270	2,276	383	7
SHIP	1,743	4	42	287	639	575	192	4
TARFS	561	6	17	80	131	207	109	11
TOYAMA	4,303	7	10	103	695	2,040	1,405	43
ULSAM	1,728	2	2	70	186	959	495	14
WCWC	551	-	-	11	111	277	148	4
ZUTE	401	9	85	173	121	13	-	-
MVP	147,356	2,698	9,457	29,552	46,200	41,461	15,176	2,812
EPICCVD	20,985	139	503	2,306	5,524	9,400	2,942	171
UKBIOBANK	350,193	809	5,232	33,895	100,351	165,603	43,124	1,179

Data are number of participants.

Table S3: Characteristics of participants by categories of eGFR

	Categories of eGFR (mL/min/1.73 m^2)								
	**<15**	**15-<30**	**30-<45**	**45-<60**	**60-<75**	**75-<90**	**90-<105**	**105-<120**	**>120**
Age	60.2 (4.7)	65.4 (4.4)	66.7 (3.6)	65.6 (3.7)	62.2 (4.1)	59.2 (4.5)	55.7 (4.3)	47 (3.5)	41.6 (3.2)
Sex									
Men	271	609	2,552	13,919	55,794	114,660	133,631	37,506	2,899
Women	58	193	1,395	9,945	40,710	82,069	115,804	33,882	2,238
Creatinine	7.1 (1.7)	2.6 (0.3)	1.7 (0.1)	1.3 (0.1)	1.1 (0.1)	0.9 (0.1)	0.8 (0.1)	0.8 (0.1)	0.6 (0.1)
Incident CHD events	90	142	523	2,693	8,303	13,614	14,619	2,706	168
Incident stroke events	8	34	214	1,087	3,186	4,793	5,298	1,002	71
BMI, kg/m2	26.4 (2.4)	27.4 (2.7)	27.7 (2.5)	27.8 (2.4)	27.8 (2.3)	27.5 (2.4)	27.3 (2.5)	27.3 (2.7)	27.1 (3)
SBP, mmHg	137 (11)	138 (10)	137 (10)	135 (9)	134 (9)	132 (8)	131 (8)	127 (8)	126 (8)
Smoking status									
Not current	303	730	3,567	21,245	84,160	169,933	212,106	56,731	3,939
Current	26	72	380	2,619	12,344	26,796	37,329	14,657	1198
HDL cholesterol, mmol/L	1.2 (0.2)	1.2 (0.2)	1.3 (0.2)	1.3 (0.2)	1.3 (0.2)	1.3 (0.2)	1.3 (0.2)	1.3 (0.2)	1.4 (0.2)
Total cholesterol, mmol/L	5.0 (0.5)	5.0 (0.6)	5.2 (0.6)	5.3 (0.5)	5.3 (0.5)	5.3 (0.5)	5.3 (0.5)	5.1 (0.5)	5.0 (0.5)

Data are n, or mean (SD). Participants with missing information on age, sex systolic blood pressure, total and high-density lipoprotein cholesterol, body-mass index, and smoking status were excluded. CHD= coronary heart disease. BMI= body-mass index. SBP= systolic blood pressure. HDL= high-density lipoprotein. eGFR= estimated glomerula filtration rate

Table S4: Variants used to make the genetic risk score for eGFR (using creatinine in the CKD-EPI equation).

RSID	Chromosome and base pair	Effect allele	Other allele	European ancestry		Trans-ancestry	
				Effect size (beta)	SE	Effect size (beta)	SE
rs11166440	1:100808363	A	G	0.0021	0.0004	0.002	0.0003
rs74748843	1:10730910	C	T	0.006	0.0013	0.0048	0.0008
rs12736457	1:113258293	C	G	0.0056	0.0005	0.0054	0.0005
rs3118119	1:150159616	T	C	0.0031	0.0005	0.003	0.0005
rs267738	1:150940625	G	T	0.005	0.0004	0.0048	0.0004
rs10159261	1:15912987	G	T	0.0038	0.0004	0.0034	0.0003
rs3845534	1:163738950	G	A	0.0019	0.0003	0.0019	0.0003
rs4656220	1:170649277	T	C	0.0021	0.0004	0.002	0.0003
rs1011731	1:172346548	G	A	0.0019	0.0003	0.0019	0.0003
rs3795503	1:180905694	T	C	0.0022	0.0004	0.002	0.0003
rs78444298	1:184672098	G	A	0.0107	0.0014	0.0105	0.0014
rs78329830	1:186769572	G	A	0.0051	0.001	0.0054	0.0009
rs12061708	1:18809916	G	A	0.0027	0.0004	0.0026	0.0003
rs3850625	1:201016296	A	G	0.0048	0.0006	0.0046	0.0005
rs2808454	1:207231751	A	T	0.0019	0.0003	0.0019	0.0003
rs75625374	1:208039431	C	G	0.0043	0.0007	0.0045	0.0007
rs7535253	1:214744893	T	C	0.0023	0.0004	0.0021	0.0004
rs2577134	1:220224321	T	C	0.0021	0.0004	0.002	0.0003
rs61830291	1:221001142	C	A	0.0036	0.0006	0.0036	0.0006
rs417237	1:228532195	T	G	0.002	0.0004	0.0018	0.0003
rs2749153	1:23699340	G	A	0.003	0.0004	0.0033	0.0003
rs2490391	1:243469669	C	A	0.0025	0.0003	0.0024	0.0003
rs688540	1:48002447	G	A	0.0031	0.0006	0.003	0.0005
rs17413465	1:55718708	A	C	0.0025	0.0004	0.0025	0.0004
rs1757915	1:56615809	A	G	0.002	0.0004	0.0021	0.0003
rs7536433	1:78023173	T	C	0.0018	0.0004	0.0021	0.0004
rs679843	1:78707493	T	C	0.002	0.0004	0.0021	0.0003
rs17050272	2:121306440	G	A	0.0022	0.0004	0.0022	0.0003
rs11694902	2:121988884	A	G	0.0041	0.0005	0.0041	0.0005
rs7425436	2:148759656	A	G	0.0026	0.0004	0.0024	0.0003
rs4664475	2:152387553	C	T	0.002	0.0004	0.002	0.0003
rs807624	2:15782471	T	G	0.0034	0.0004	0.0032	0.0003
rs35472707	2:169995581	C	T	0.0075	0.0008	0.0073	0.0008
rs187355703	2:176993583	C	G	0.0101	0.0011	0.01	0.0011
rs35284526	2:178121524	A	C	0.0029	0.0004	0.0029	0.0003
rs4666821	2:183077254	T	G	0.0018	0.0003	0.002	0.0003
rs4491726	2:18676276	A	G	0.0032	0.0004	0.0032	0.0004
rs60980181	2:188168567	T	A	0.0029	0.0005	0.0027	0.0004
rs1047891	2:211540507	C	A	0.0065	0.0004	0.0065	0.0004
rs1548945	2:217665788	T	C	0.0037	0.0004	0.0036	0.0003
rs1050816	2:220358198	T	C	0.0029	0.0004	0.0026	0.0003
rs35669853	2:227287718	A	G	0.0026	0.0004	0.0024	0.0004
rs13003198	2:234257105	T	C	0.0017	0.0004	0.0018	0.0003
rs2301343	2:40680149	G	T	0.0023	0.0004	0.0023	0.0004
rs10865189	2:43433257	C	G	0.0025	0.0004	0.0024	0.0003
rs2971880	2:54885640	T	A	0.0026	0.0004	0.0024	0.0003
rs10197255	2:67874553	A	T	0.0018	0.0004	0.0018	0.0003
rs6546869	2:73895765	A	G	0.0061	0.0004	0.0059	0.0004

RSID	Chromosome and base pair	Effect allele	Other allele	European ancestry		Trans-ancestry	
				Effect size (beta)	SE	Effect size (beta)	SE
rs2289746	3:105455955	C	T	0.0016	0.0004	0.0019	0.0003
rs9868185	3:121657593	A	G	0.0027	0.0003	0.0026	0.0003
rs10934754	3:125906237	T	C	0.0017	0.0003	0.002	0.0003
rs35320690	3:135932494	C	T	0.0025	0.0004	0.0025	0.0004
rs9828976	3:136536835	G	C	0.0024	0.0004	0.0024	0.0004
rs7624084	3:141093285	T	C	0.0015	0.0003	0.0017	0.0003
rs1397764	3:141750810	A	G	0.0047	0.0004	0.0043	0.0003
rs76272256	3:168888112	T	C	0.0024	0.0005	0.0024	0.0004
rs56065557	3:185354216	G	C	0.003	0.0004	0.0029	0.0003
rs9823161	3:193811168	A	G	0.0021	0.0004	0.0022	0.0004
rs6779998	3:30749965	G	A	0.0019	0.0003	0.0017	0.0003
rs3774726	3:63974477	C	T	0.0023	0.0004	0.0021	0.0003
rs3775932	4:10090930	C	A	0.002	0.0003	0.0018	0.0003
rs223471	4:103698786	C	G	0.0028	0.0004	0.0028	0.0003
rs55929207	4:109703549	C	G	0.002	0.0003	0.0019	0.0003
rs16874073	4:23743962	C	T	0.0041	0.0008	0.0045	0.0007
rs75501914	4:3449781	A	G	0.0042	0.0008	0.0039	0.0006
rs4864890	4:52686513	C	T	0.0023	0.0004	0.0023	0.0004
rs12509595	4:81182554	C	T	0.0032	0.0004	0.0035	0.0003
rs12777	5:131671662	C	G	0.005	0.0009	0.005	0.0009
rs11743174	5:148524820	T	C	0.0019	0.0004	0.0019	0.0003
rs3812036	5:176813404	C	T	0.0069	0.0004	0.0065	0.0004
rs13157326	5:34504277	G	A	0.0027	0.0004	0.0027	0.0003
rs495237	5:39950266	T	G	0.0029	0.0004	0.0027	0.0003
rs11746506	5:44812566	T	C	0.0018	0.0004	0.0017	0.0003
rs79760705	5:53298716	T	G	0.0056	0.0006	0.0056	0.0005
rs72759880	5:67750213	G	T	0.0057	0.0005	0.0056	0.0005
rs2010352	5:68656327	G	A	0.0019	0.0003	0.0018	0.0003
rs3797537	5:78322650	A	G	0.0021	0.0004	0.0019	0.0003
rs1857859	6:100894587	A	G	0.0014	0.0004	0.0019	0.0003
rs1268168	6:109008158	A	G	0.0027	0.0004	0.0024	0.0003
rs7740107	6:130374461	A	T	0.0027	0.0004	0.0027	0.0004
rs9375818	6:131882078	G	A	0.0026	0.0004	0.0031	0.0004
rs3822939	6:133849789	G	A	0.0028	0.0003	0.0025	0.0003
rs9397738	6:154986664	A	G	0.0025	0.0005	0.0027	0.0004
rs12207180	6:160633107	T	A	0.0085	0.0005	0.0085	0.0005
rs3765502	6:24354045	T	C	0.0017	0.0006	0.0024	0.0004
rs144100226	6:34180297	T	C	0.006	0.0011	0.0059	0.001
rs13200335	6:41690823	A	C	0.0024	0.0003	0.0024	0.0003
rs77915916	6:43287722	A	T	0.0047	0.0006	0.0046	0.0006
rs881858	6:43806609	G	A	0.0056	0.0004	0.0054	0.0003
rs720989	6:44765535	T	G	0.0023	0.0004	0.0021	0.0004
rs6458868	6:52630153	C	T	0.0021	0.0004	0.002	0.0003
rs3925003	6:55422618	C	T	0.0019	0.0003	0.0018	0.0003
rs11755724	6:7118990	A	G	0.0027	0.0004	0.0027	0.0004
rs72912510	6:90118764	G	A	0.002	0.0004	0.0024	0.0004
rs3757387	7:128576086	T	C	0.0029	0.0004	0.003	0.0003
rs62435145	7:1286567	G	T	0.0055	0.0004	0.006	0.0004
rs62491533	7:129564134	C	T	0.0027	0.0005	0.0027	0.0004
rs10254101	7:151415536	C	T	0.0068	0.0004	0.0068	0.0004
rs12671694	7:155665959	T	C	0.0028	0.0004	0.0025	0.0003

RSID	Chromosome and base pair	Effect allele	Other allele	European ancestry		Trans-ancestry	
				Effect size (beta)	SE	Effect size (beta)	SE
rs868822	7:156252939	T	G	0.0032	0.0004	0.0029	0.0003
rs6968554	7:17287106	G	A	0.0022	0.0004	0.0019	0.0003
rs3750081	7:32930876	G	T	0.0019	0.0004	0.0022	0.0003
rs55773927	7:65337902	T	C	0.002	0.0003	0.0019	0.0003
rs41301394	7:75612803	T	C	0.002	0.0004	0.0023	0.0003
rs11783418	8:10841858	G	A	0.002	0.0004	0.002	0.0004
rs10098664	8:11417493	C	T	0.0024	0.0004	0.0021	0.0003
rs2954017	8:126476873	T	C	0.0026	0.0004	0.0024	0.0003
rs34861762	8:23748420	C	T	0.0043	0.0003	0.0043	0.0003
rs10102889	8:32435620	G	C	0.005	0.0013	0.0036	0.0006
rs2980423	8:8142575	C	T	0.0025	0.0004	0.0023	0.0003
rs1533059	8:8684953	A	G	0.0028	0.0004	0.0025	0.0003
rs2976178	8:87332552	G	C	0.0027	0.0004	0.0025	0.0003
rs35353426	8:9297246	C	T	0.0026	0.0004	0.0026	0.0004
rs1321917	9:119324929	G	C	0.0025	0.0003	0.0023	0.0003
rs7024579	9:139100413	T	C	0.0023	0.0004	0.0023	0.0004
rs28404308	9:140103272	A	T	0.0027	0.0005	0.0024	0.0004
rs12377027	9:20554583	G	A	0.0027	0.0005	0.0026	0.0005
rs13287724	9:33169034	T	A	0.0029	0.0006	0.003	0.0006
rs544169	9:33956791	A	G	0.0024	0.0004	0.0022	0.0003
rs284859	10:104573017	T	G	0.0027	0.0004	0.0026	0.0004
rs1536225	10:105202318	G	T	0.0019	0.0004	0.0021	0.0003
rs6481598	10:29781798	C	G	0.0023	0.0004	0.0024	0.0004
rs7072591	10:35150364	A	G	0.0019	0.0004	0.0019	0.0003
rs8474	10:51026705	C	G	0.0019	0.0003	0.002	0.0003
rs10821905	10:52646093	A	G	0.0039	0.0004	0.0037	0.0004
rs7475348	10:69965177	T	C	0.0027	0.0004	0.0031	0.0003
rs12240572	10:75016365	T	A	0.0034	0.0007	0.0032	0.0006
rs816850	10:79252446	G	C	0.002	0.0004	0.002	0.0004
rs7095954	10:82209232	T	A	0.0019	0.0003	0.0018	0.0003
rs9420446	10:88880689	T	C	0.0022	0.0005	0.0023	0.0004
rs80282103	10:899071	A	T	0.0081	0.0006	0.0078	0.0006
rs2068888	10:94839642	G	A	0.0026	0.0003	0.0024	0.0003
rs4918943	10:97278922	G	A	0.0023	0.0005	0.0022	0.0004
rs6589750	11:119326726	A	G	0.0017	0.0004	0.002	0.0003
rs10790452	11:121584931	T	C	0.002	0.0004	0.002	0.0003
rs11564722	11:2178330	T	C	0.0038	0.0004	0.0033	0.0004
rs63934	11:2789062	A	G	0.0042	0.0004	0.0041	0.0004
rs963837	11:30749090	C	T	0.0055	0.0004	0.0057	0.0003
rs61897431	11:47427667	T	C	0.0028	0.0004	0.0029	0.0004
rs7127946	11:48250675	T	C	0.0023	0.0004	0.0023	0.0003
rs2727040	11:49057603	C	T	0.0026	0.0006	0.0026	0.0004
rs1813937	11:50468801	T	C	0.0019	0.0004	0.0022	0.0004
rs1541937	11:5578558	C	A	0.0029	0.0004	0.0029	0.0004
rs1783827	11:57409538	G	A	0.0021	0.0004	0.002	0.0003
rs948493	11:65552154	C	T	0.0032	0.0004	0.0033	0.0003
rs11237450	11:78023356	A	C	0.003	0.0005	0.0032	0.0004
rs117113238	12:12209203	A	G	0.0039	0.0006	0.0039	0.0006
rs10846157	12:15325031	C	A	0.0036	0.0004	0.0034	0.0004
rs632887	12:3392351	A	G	0.0033	0.0004	0.0032	0.0003
rs11062167	12:364739	G	A	0.0042	0.0003	0.0039	0.0003

RSID	Chromosome and base pair	Effect allele	Other allele	European ancestry		Trans-ancestry	
				Effect size (beta)	SE	Effect size (beta)	SE
rs4238020	12:4616642	T	C	0.0028	0.0005	0.0029	0.0005
rs2634675	12:48740855	A	G	0.0028	0.0004	0.0025	0.0003
rs12313306	12:57751854	T	C	0.0031	0.0004	0.0029	0.0004
rs41284816	13:50655989	G	T	0.0079	0.0012	0.0078	0.0012
rs500830	13:72348768	T	C	0.0026	0.0003	0.0029	0.0003
rs61993680	14:100752644	C	A	0.0022	0.0004	0.0019	0.0003
rs72683923	14:50735947	C	T	0.0076	0.0014	0.0074	0.0013
rs6574652	14:81870100	C	T	0.0019	0.0003	0.0017	0.0003
rs1028455	14:88829975	A	T	0.0021	0.0004	0.002	0.0003
rs17184313	14:93102251	C	T	0.0028	0.0005	0.0029	0.0005
rs12913015	15:39305443	T	C	0.0028	0.0004	0.0027	0.0003
rs6492982	15:41399951	C	T	0.0032	0.0004	0.0033	0.0004
rs1994887	15:57793765	C	A	0.0024	0.0004	0.002	0.0004
rs956006	15:62808539	T	C	0.0022	0.0004	0.0019	0.0003
rs11071738	15:63580155	C	T	0.0025	0.0003	0.0025	0.0003
rs11071939	15:67463391	C	T	0.0038	0.0007	0.0039	0.0006
rs351237	15:74477239	G	A	0.0018	0.0004	0.0018	0.0003
rs2472297	15:75027880	T	C	0.0039	0.0004	0.0039	0.0004
rs4886696	15:75664570	T	A	0.0033	0.0004	0.0032	0.0004
rs4886755	15:76298132	A	G	0.0041	0.0003	0.0041	0.0003
rs166906	15:76802175	T	C	0.0039	0.0006	0.0033	0.0005
rs17507300	15:83722059	A	G	0.0024	0.0005	0.0024	0.0004
rs7169629	15:85191274	C	G	0.0019	0.0003	0.0018	0.0003
rs59646751	15:99276521	G	T	0.002	0.0004	0.0023	0.0003
rs193538	16:16127916	G	T	0.002	0.0004	0.002	0.0003
rs438339	16:2003425	T	C	0.0035	0.0007	0.0035	0.0006
rs77924615	16:20392332	A	G	0.0096	0.0005	0.0098	0.0004
rs1635404	16:3747042	G	T	0.0024	0.0004	0.0025	0.0004
rs9932625	16:51735746	G	A	0.003	0.0004	0.003	0.0003
rs7203398	16:53189672	A	C	0.0027	0.0004	0.0025	0.0003
rs7185391	16:68323115	G	T	0.0026	0.0004	0.0027	0.0004
rs62053077	16:71643669	G	T	0.0025	0.0004	0.0021	0.0004
rs1858800	16:73024276	T	C	0.0022	0.0004	0.002	0.0003
rs154656	16:89708003	T	A	0.0031	0.0003	0.003	0.0003
rs28735420	17:12139964	T	G	0.004	0.0008	0.0039	0.0006
rs2349648	17:17017267	G	T	0.0022	0.0004	0.0017	0.0003
rs9891340	17:17543846	T	C	0.0024	0.0004	0.0024	0.0004
rs2440165	17:19428719	T	C	0.0041	0.0004	0.004	0.0003
rs2411192	17:34882998	T	A	0.0024	0.0003	0.0024	0.0003
rs227731	17:54773238	T	G	0.0018	0.0004	0.0018	0.0003
rs35662455	17:56755223	C	G	0.003	0.0005	0.003	0.0005
rs9903801	17:58915261	C	G	0.0049	0.0005	0.0047	0.0004
rs9895661	17:59456589	T	C	0.0074	0.0005	0.0069	0.0004
rs8866	17:65373979	G	C	0.0018	0.0004	0.0018	0.0003
rs883541	17:66449122	G	A	0.0023	0.0004	0.0022	0.0003
rs1719934	18:5585158	A	G	0.0028	0.0003	0.0026	0.0003
rs2974751	19:13053034	A	C	0.0019	0.0004	0.0018	0.0003
rs7251730	19:36997147	T	C	0.0024	0.0004	0.0024	0.0003
rs78241494	19:37649748	C	T	0.0031	0.0004	0.003	0.0004
rs113445505	19:38157969	T	C	0.0038	0.0004	0.0037	0.0003
rs34647824	19:50138143	C	A	0.002	0.0004	0.0021	0.0004

RSID	Chromosome and base pair	Effect allele	Other allele	European ancestry		Trans-ancestry	
				Effect size (beta)	SE	Effect size (beta)	SE
rs62187537	20:1333060	T	C	0.0038	0.0007	0.0039	0.0007
rs1041606	20:14677788	C	T	0.002	0.0004	0.0021	0.0004
rs6087579	20:32985155	G	A	0.003	0.0003	0.0028	0.0003
rs2273684	20:33529766	T	G	0.0033	0.0003	0.0032	0.0003
rs17216707	20:52732362	C	T	0.0052	0.0005	0.0051	0.0004
rs2235826	20:56143169	T	A	0.0033	0.0005	0.003	0.0004
rs72629024	20:62152519	C	G	0.0034	0.0006	0.0035	0.0005
rs4408777	20:62706105	G	A	0.0021	0.0004	0.0021	0.0003
rs1509117	20:8303120	A	T	0.0025	0.0004	0.0024	0.0004
rs2823139	21:16576783	G	A	0.0027	0.0004	0.0026	0.0003
rs2834317	21:35356706	G	A	0.0031	0.0005	0.0035	0.0005
rs2244237	21:37818141	T	G	0.0027	0.0004	0.0027	0.0004
rs131263	22:30133045	T	C	0.0023	0.0004	0.0024	0.0004
rs80576	22:36539804	G	A	0.0027	0.0005	0.0028	0.0005
rs4820324	22:38599857	G	C	0.0023	0.0004	0.0023	0.0003
rs112880707	22:40884662	T	C	0.0056	0.0006	0.0052	0.0005
rs738527	22:43112961	T	C	0.0031	0.0004	0.0032	0.0003

SE= standard error. Weights were taken from the discovery GWAS of the CKDGen study.[20]

Table S5: Pairwise Pearson correlations of GRSs for eGFR and body-mass index, by study

	Creatinine-based eGFR	BMI	GRS	GRS (Cys)	GRS (BUN)
UKB					
GRS	0.1730*	-0.0037			
GRS (Cys)	0.1447*	-0.0031	0.8003*		
GRS (BUN)	0.1378*	0.0009	0.7626*	0.7661*	
GRS (Raw)	0.2005*	-0.0039	0.8898*	0.7062*	0.6731*
EPIC-CVD					
GRS	0.1357*	-0.0203			
GRS (Cys)	0.1183*	-0.0037	0.7954*		
GRS (BUN)	0.1025*	-0.0199	0.7591*	0.7564*	
GRS (Raw)	0.1598*	-0.0079	0.8851*	0.6972*	0.6612*
MVP					
GRS	0.1475*	-0.0021			
GRS (Cys)	0.1267*	0.0029	0.8032*		
GRS (BUN)	0.1240*	-0.0034	0.6507*	0.6626*	
GRS (Raw)	0.1670*	-0.0058	0.8894*	0.7090*	0.6934*

GRS was constructed using 218 European-specific eGFR (creatinine-based) associated genetic variants (n=567,460). GRS (Cys) included 127 genetic variants (out of the 218 genetic variants included in the GRS for eGFR) that were additionally associated with cystatin-C-based eGFR (n=460,826). GRS (BUN) included 121 genetic variants (out of the 218 genetic variants included in the GRS for eGFR) that were additionally associated with blood urine nitrogen (n=416,178). GRS (Raw) included all the 262 eGFR-associated index variants in CKDGen trans-ancestry analysis (n=765,348). * Bonferroni-corrected significance (P<0.01). GRS= genetic risk score. eGFR= estimated glomerular filtration rate. BMI= body-mass index.

Table S6: Mendelian randomization estimates of each 5 mL/min/1.73 m^2 lower genetically-predicted eGFR with risk of coronary heart disease and stroke

eGFR within studies, mL/min/1.73m2	Mean eGFR, mL/min/1.73m2	No. of participants	Coronary heart disease		Stroke	
			No. of events	HR (95% CI)	No. of events	HR (95% CI)
**EPIC-CVD**						
< 60	50.1	533	266	1.52 (1.09, 2.13)	103	2.30 (1.32, 4.02)
60 - 74	69.1	1979	847	1.10 (0.94, 1.29)	322	1.13 (0.86, 1.49)
75 - 89	83.3	4953	1760	1.08 (0.97, 1.21)	777	1.01 (0.85, 1.20)
90 - 104	97.6	8787	2619	1.11 (1.00, 1.23)	1382	1.01 (0.88, 1.16)
≥105*	111.0	3569	658	0.85 (0.71, 1.02)	376	0.83 (0.64, 1.09)
**MVP**						
< 60	50.6	9241	1146	1.18 (1.04, 1.34)	250	1.10 (0.84, 1.44)
60 - 74	68.3	22,916	2008	1.06 (0.97, 1.16)	484	1.17 (0.97, 1.41)
75 - 89	82.9	34,890	2752	1.05 (0.97, 1.14)	587	0.92 (0.78, 1.09)
90 - 104	96.4	30,130	2285	0.94 (0.86, 1.02)	514	1.10 (0.91, 1.33)
≥105*	111.7	8085	316	0.98 (0.77, 1.24)	79	1.41 (0.87, 2.27)
**UK Biobank**						
< 60	52.4	5044	337	0.84 (0.66, 1.08)	138	1.03 (0.70, 1.53)
60 - 74	69.4	28,361	1459	1.10 (0.97, 1.23)	512	1.11 (0.91, 1.34)
75 - 89	83.5	83,821	3717	1.04 (0.97, 1.11)	1201	0.98 (0.87, 1.11)
90 - 104	97.0	137,263	5069	1.00 (0.94, 1.07)	1686	1.07 (0.96, 1.20)
≥105*	109.1	34,146	678	0.94 (0.79, 1.11)	211	0.97 (0.71, 1.31)
**Combined**						
< 60	51.2	14,818	1749	1.14 (1.03, 1.27)	491	1.19 (0.97, 1.47)
60 - 74	68.9	53,256	4314	1.08 (1.01, 1.15)	1318	1.14 (1.01, 1.28)
75 - 89	83.3	123,664	8229	1.05 (1.00, 1.10)	2565	0.97 (0.89, 1.06)
90 - 104	96.9	176,180	9973	1.01 (0.96, 1.05)	3582	1.06 (0.98, 1.14)
≥105*	109.7	45,800	1652	0.91 (0.82, 1.02)	666	0.95 (0.79, 1.14)

HRs are shown per 5 mL/min/1.73 m^2 lower genetically-predicted eGFR and are adjusted for age, age-squared, sex, study center, and the first ten principal components. *HRs in the group with eGFR above 105 were shown per 5 mL/min/1.73 m^2 *higher* genetically-predicted eGFR. Mean eGFR within each stratum was weighted by the number of participants from each contributing study, and MR estimates within each stratum were meta-analyzed using inverse variance weighting and fixed effects.

Table S7: Comparison of estimates from residual and doubly-ranked non-linear Mendelian randomization methods.

Million Veteran Program (MVP)					
	Residual method		Doubly-ranked method		
Stratum	eGFR	HR (95% CI)	eGFR	Genetic association with eGFR	HR (95% CI)
1	51.9 (46.0, 61.2)	1.19 (1.04, 1.36)	55.8 (47.0, 70.1)	2.39	1.15 (1.01, 1.31)
2	65.5 (61.3, 69.8)	1.15 (1.01, 1.32)	66.3 (55.2, 77.3)	2.78	1.10 (0.97, 1.24)
3	71.9 (68.1, 75.9)	0.95 (0.83, 1.10)	72.5 (62.4, 82.9)	2.98	1.18 (1.05, 1.33)
4	77.2 (73.6, 80.7)	1.06 (0.92, 1.23)	77.4 (67.7, 87.5)	3.02	1.00 (0.89, 1.13)
5	82.0 (78.1, 86.2)	1.07 (0.94, 1.23)	81.9 (72.1, 90.9)	2.95	0.90 (0.80, 1.02)
6	87.0 (83.3, 90.5)	0.90 (0.78, 1.04)	86.0 (77.0, 94.1)	2.72	0.96 (0.84, 1.09)
7	90.6 (87.3, 93.8)	1.03 (0.89, 1.19)	89.9 (81.0, 97.7)	2.46	1.03 (0.89, 1.19)
8	94.4 (90.8, 97.9)	0.97 (0.84, 1.12)	94.0 (86.4, 101.9)	2.19	0.97 (0.83, 1.14)
9	99.2 (95.3, 103.3)	0.96 (0.83, 1.12)	98.9 (90.6, 107.9)	1.96	1.00 (0.82, 1.21)
10	109.5 (102.8, 114.1)	1.35 (1.09, 1.66)	106.5 (96.1, 113.4)	1.81	1.19 (0.93, 1.52)
UK Biobank					
	Residual method		Doubly-ranked method		
Stratum	eGFR	HR (95% CI)	eGFR	Genetic association with eGFR	HR (95% CI)
1	65.5 (60.4, 73.4)	1.04 (0.93, 1.16)	69.2 (61.3, 81.8)	2.75	1.06 (0.98, 1.15)
2	77.4 (73.4, 81.4)	1.07 (0.96, 1.18)	78.2 (68.0, 88.3)	2.91	0.98 (0.91, 1.06)
3	83.3 (79.7, 87.0)	0.93 (0.84, 1.02)	83.5 (74.3, 92.0)	2.86	1.00 (0.92, 1.09)
4	88.0 (84.5, 91.5)	0.94 (0.85, 1.04)	87.5 (79.1, 94.7)	2.68	0.98 (0.90, 1.07)
5	91.5 (88.3, 94.6)	0.86 (0.77, 0.95)	90.8 (83.3, 97.1)	2.40	0.94 (0.85, 1.04)
6	94.2 (91.1, 97.3)	0.98 (0.88, 1.10)	93.7 (87.2, 99.7)	2.13	0.98 (0.87, 1.10)
7	96.8 (93.6, 99.9)	1.12 (1.00, 1.26)	96.5 (90.6, 102.3)	1.91	1.02 (0.89, 1.17)
8	99.7 (96.5, 102.9)	1.30 (1.15, 1.47)	99.4 (93.5, 105.3)	1.72	0.95 (0.81, 1.12)
9	103.4 (100.0, 106.8)	1.25 (1.08, 1.44)	102.8 (96.7, 108.6)	1.52	1.24 (1.02, 1.51)
10	109.3 (104.8, 112.2)	1.18 (0.99, 1.42)	107.7 (101.0, 111.8)	1.35	1.46 (1.15, 1.85)

Mean level of eGFR in each stratum, 10th and 90th percentiles in brackets (20th and 80th percentiles for the highest and lowest decile groups); hazard ratio (HR) for each decile group per 5 mL/min/1.73 m^2 lower genetically-predicted eGFR, 95% confidence interval (CI) in brackets; genetic association with eGFR per 1 standard deviation increase in the genetic risk score.

Figure S1: Distributions of creatinine measurements by study

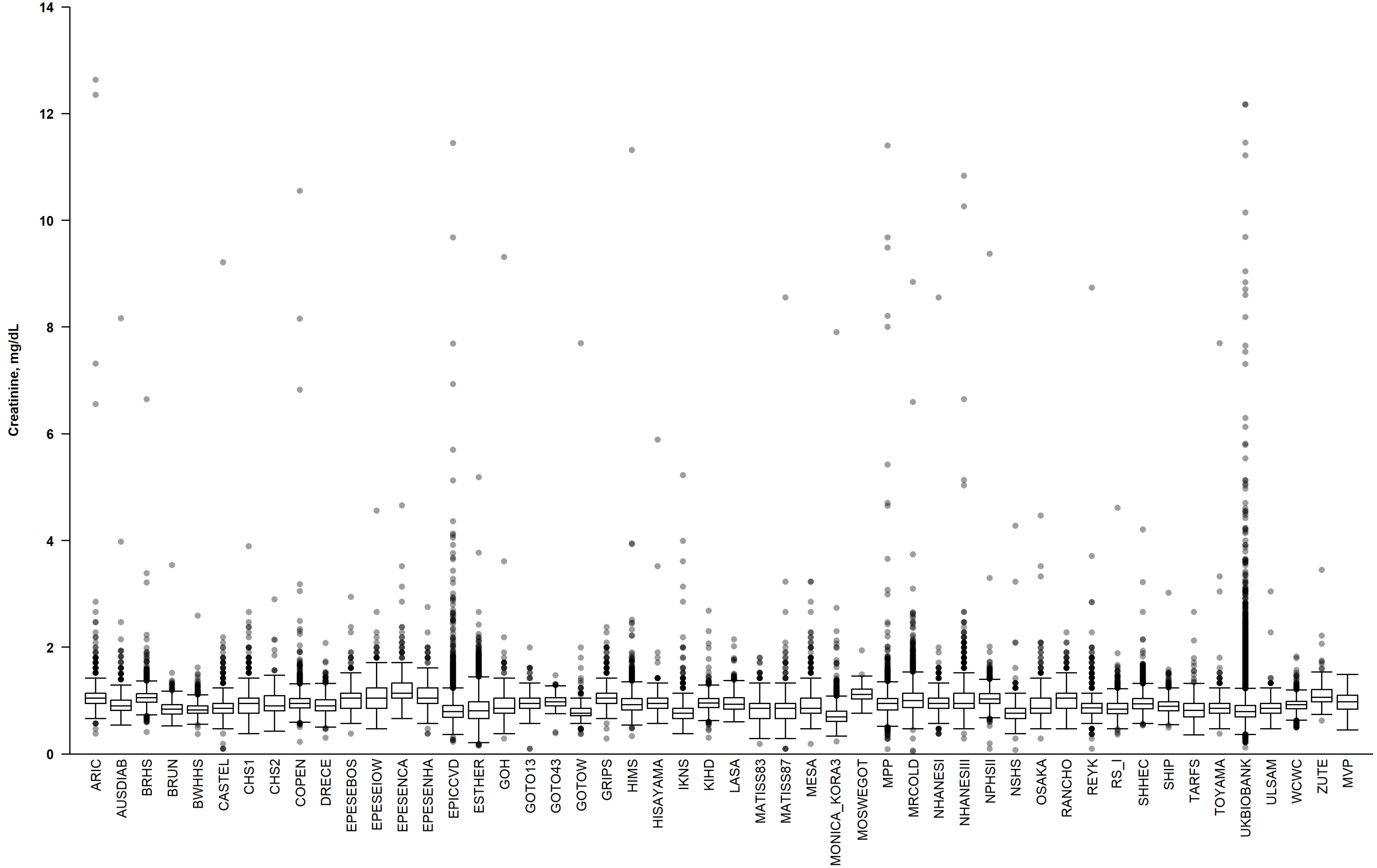

Figure S2: Distributions of measured eGFR by study

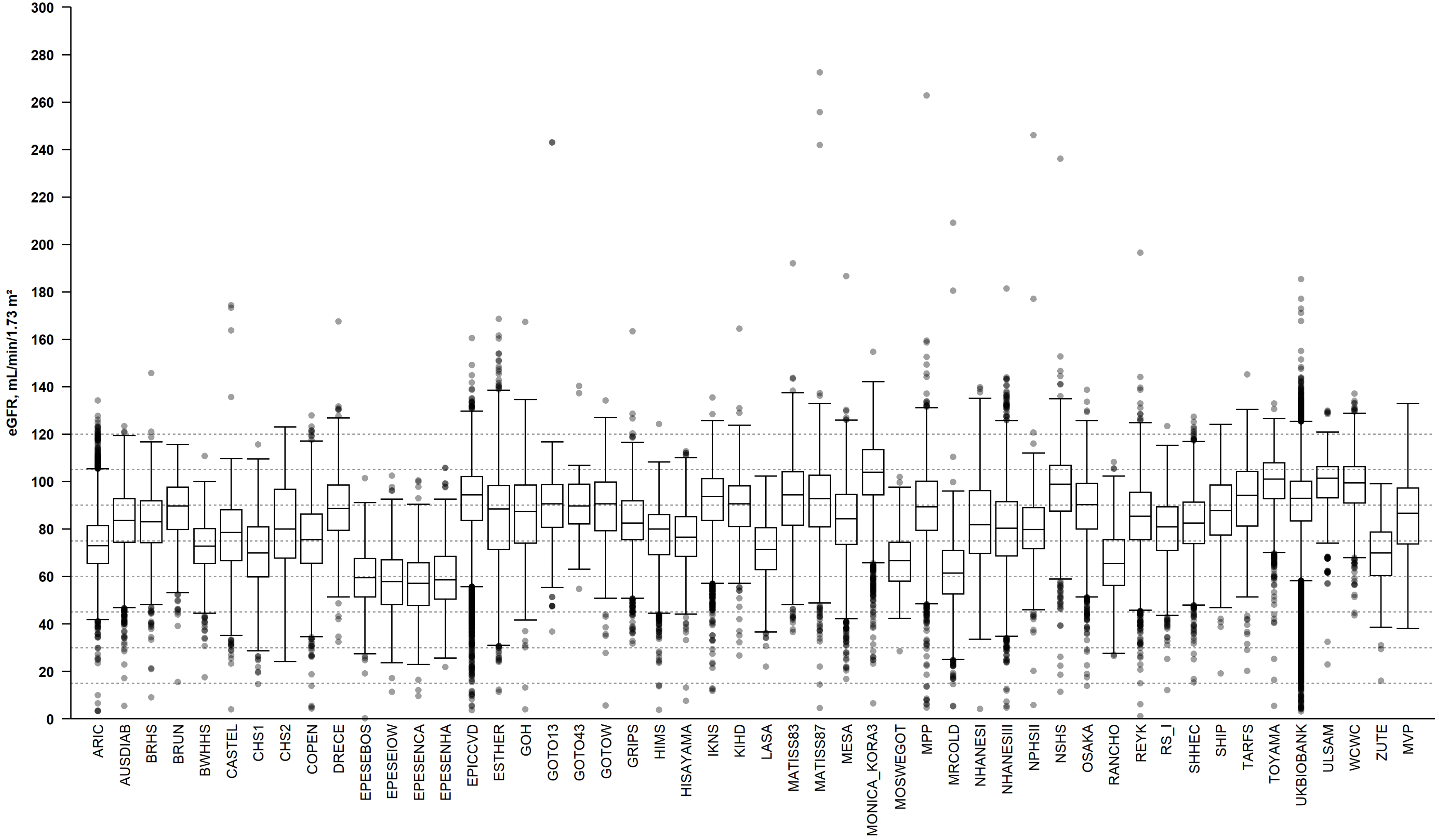

Participants with eGFR levels greater than 300 mL/min/1.73 m^2 were excluded from the analysis.

Figure S3: Observational associations of creatinine-based eGFR with risk of coronary heart disease and stroke (n=648,135)

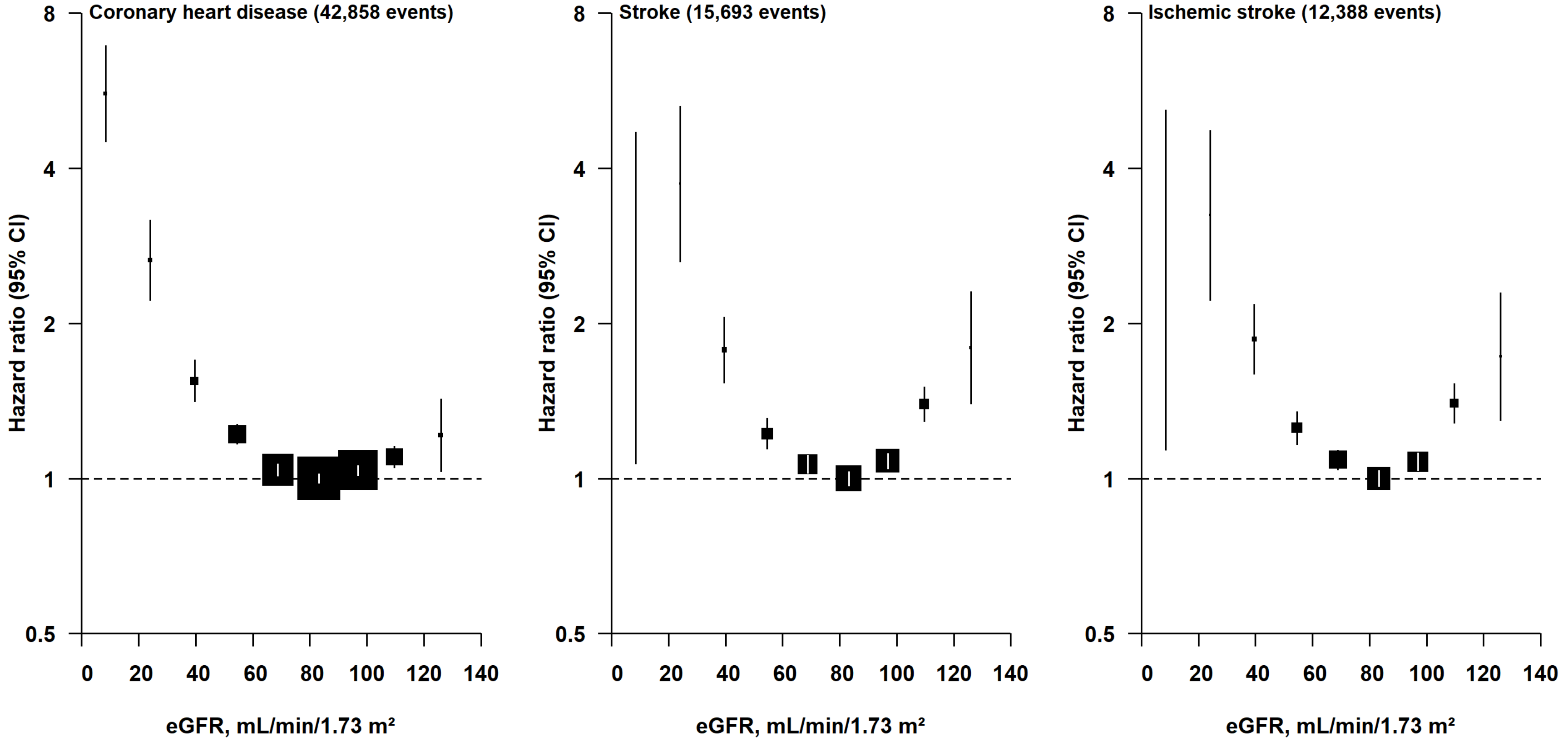

Participants with missing information on age, sex, systolic blood pressure, total and high-density lipoprotein cholesterol, body-mass index, smoking status were excluded from the analysis. Hazards ratios were estimated using Cox regression, adjusted for age, sex and study centre, where appropriate. The eGFR was estimated using creatinine-based CKD-EPI formula. The categories of eGFR are <15, 15-<30, 30-<44, 45-<60, 60-<75, 75-<90, 90-<105, 105-<120, and ≥120 mL/min/1.73 m^2. The reference category is 75-<90 mL/min/1.73 m^2. Hazards ratios were plotted against the mean eGFR in each category. Sizes of the boxes are proportional to the inverse of the variance of the log risk within that specific group. Vertical lines represented 95% confidence intervals.

Figure S4: Observational associations of creatinine-based eGFR with risk of coronary heart disease and stroke by sex, smoking, adiposity and hypertension.

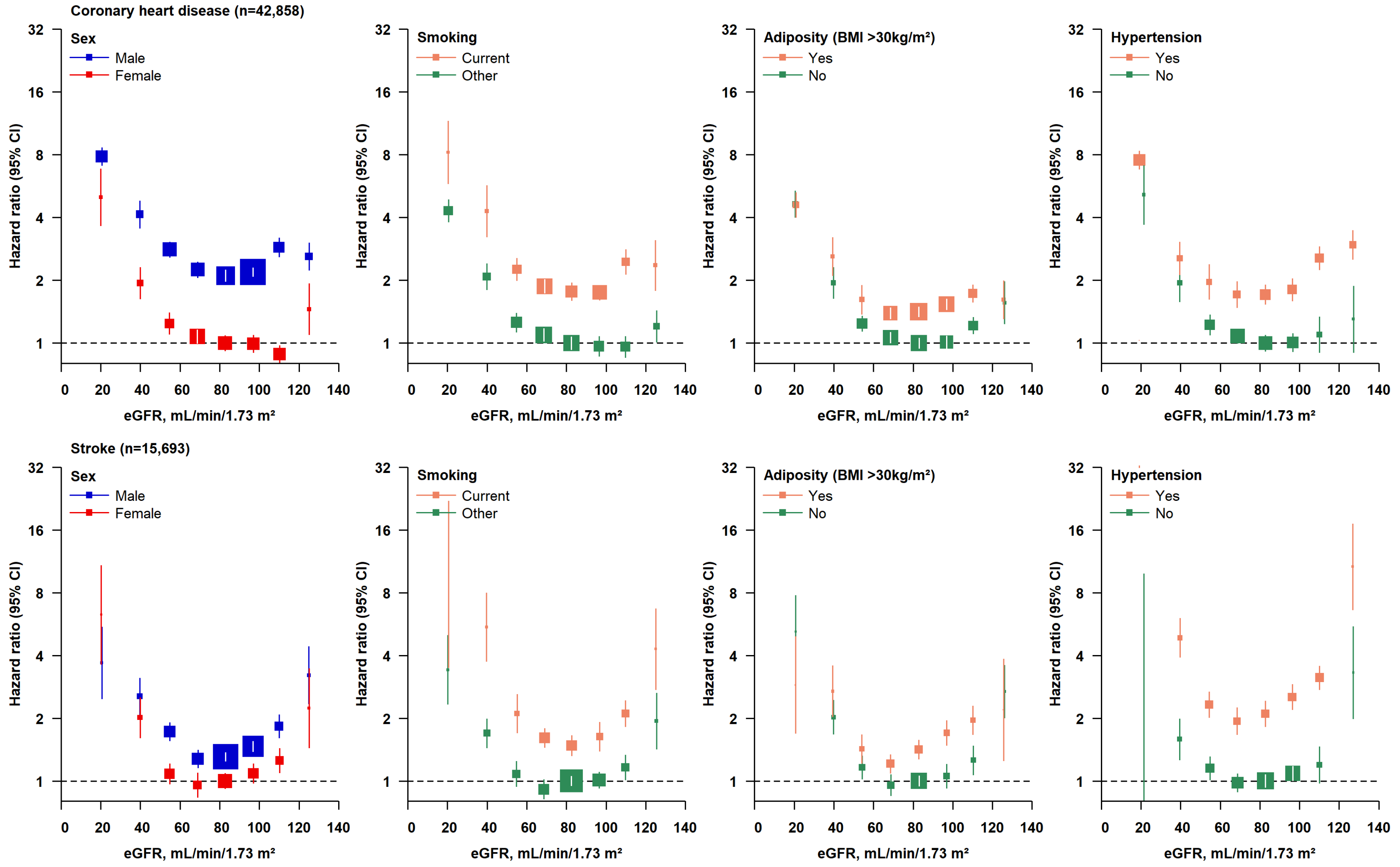

Participants with missing information on age, sex, systolic blood pressure, total and high-density lipoprotein cholesterol, body-mass index, smoking status were excluded from the analysis. Hazards ratios were estimated using Cox regression, adjusted for age, sex and study centre. The categories of eGFR are <30, 30-<44, 45-<60, 60-<75, 75-<90, 90-<105, 105-<120, and ≥120 mL/min/1.73 m^2. The reference category is 75-<90 mL/min/1.73 m^2 in females, non-smokers, participants with no history of diabetes, or hypertension in the respective panels. Hazards ratios were plotted against the mean eGFR in each category. Sizes of the boxes are proportional to the inverse of the log risk in that specific group. Vertical lines represented 95% confidence intervals. Hypertension was defined as recorded prior history of hypertension or systolic blood pressure ≥140 mm Hg and diastolic blood pressure ≥90 mm Hg.

Figure S5: Observational associations of creatinine-based eGFR with risk of coronary heart disease and stroke, by contributing study

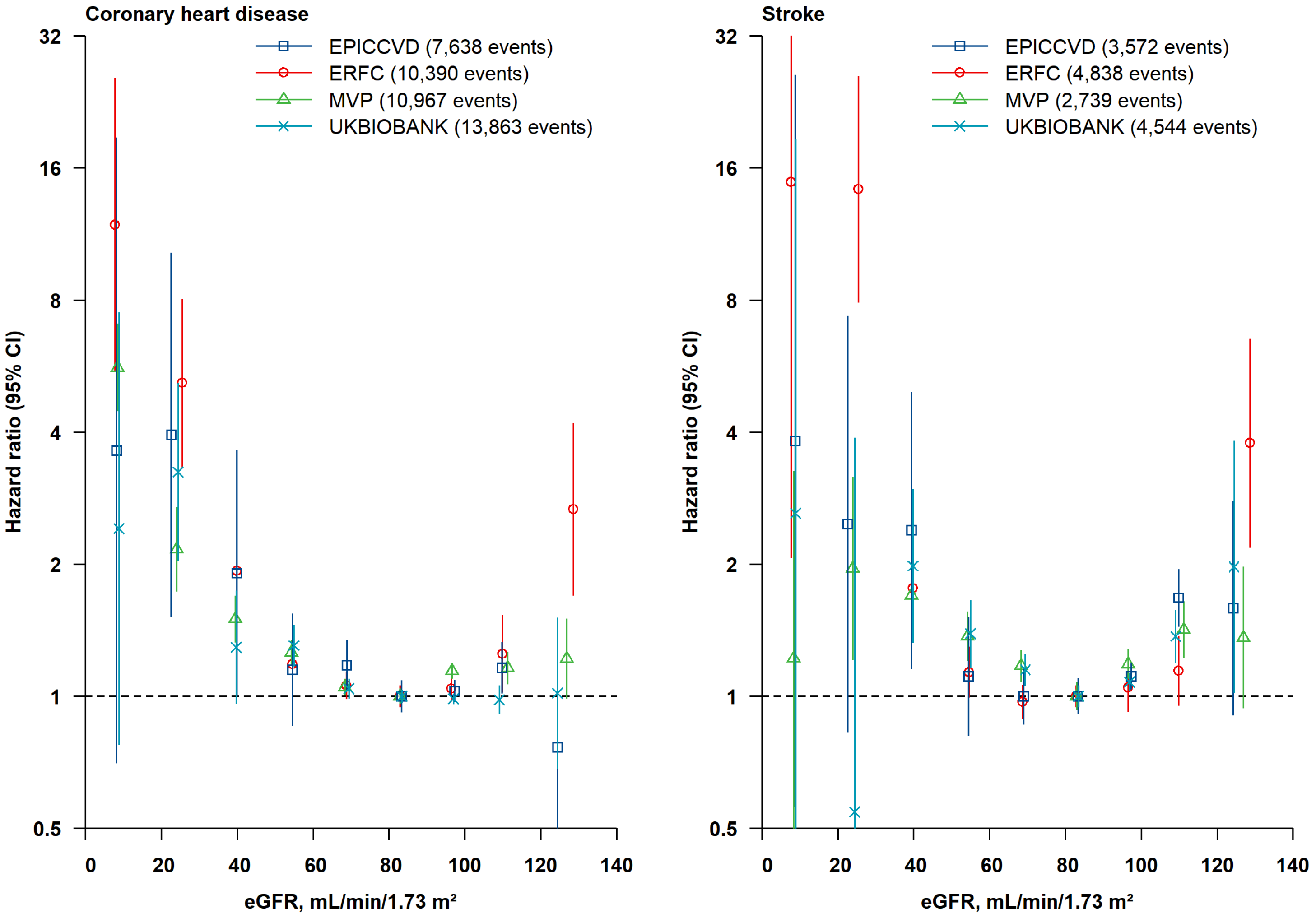

Participants with missing information on age, sex, systolic blood pressure, total and high-density lipoprotein cholesterol, body-mass index, smoking status were excluded from the analysis. Hazards ratios were estimated using Cox regression, adjusted for age, and systolic blood pressure, total and high-density lipoprotein cholesterol, body-mass index, and smoking status, and stratified by sex and study center, where appropriate. The eGFR was estimated using creatinine-based CKD-EPI formula. The categories of eGFR are <15, 15-<30, 30-<44, 45-<60, 60-<75, 75-<90, 90-<105, 105-<120, and ≥120 mL/min/1.73 m^2. The reference category is 75-<90 mL/min/1.73 m^2. Hazards ratios were plotted against the mean eGFR in each category, with vertical lines representing 95% confidence intervals.

Figure S6: Observational association of creatinine-based eGFR with risk of coronary heart disease and stroke, *irrespective of diabetes status at recruitment* (n=732,808)

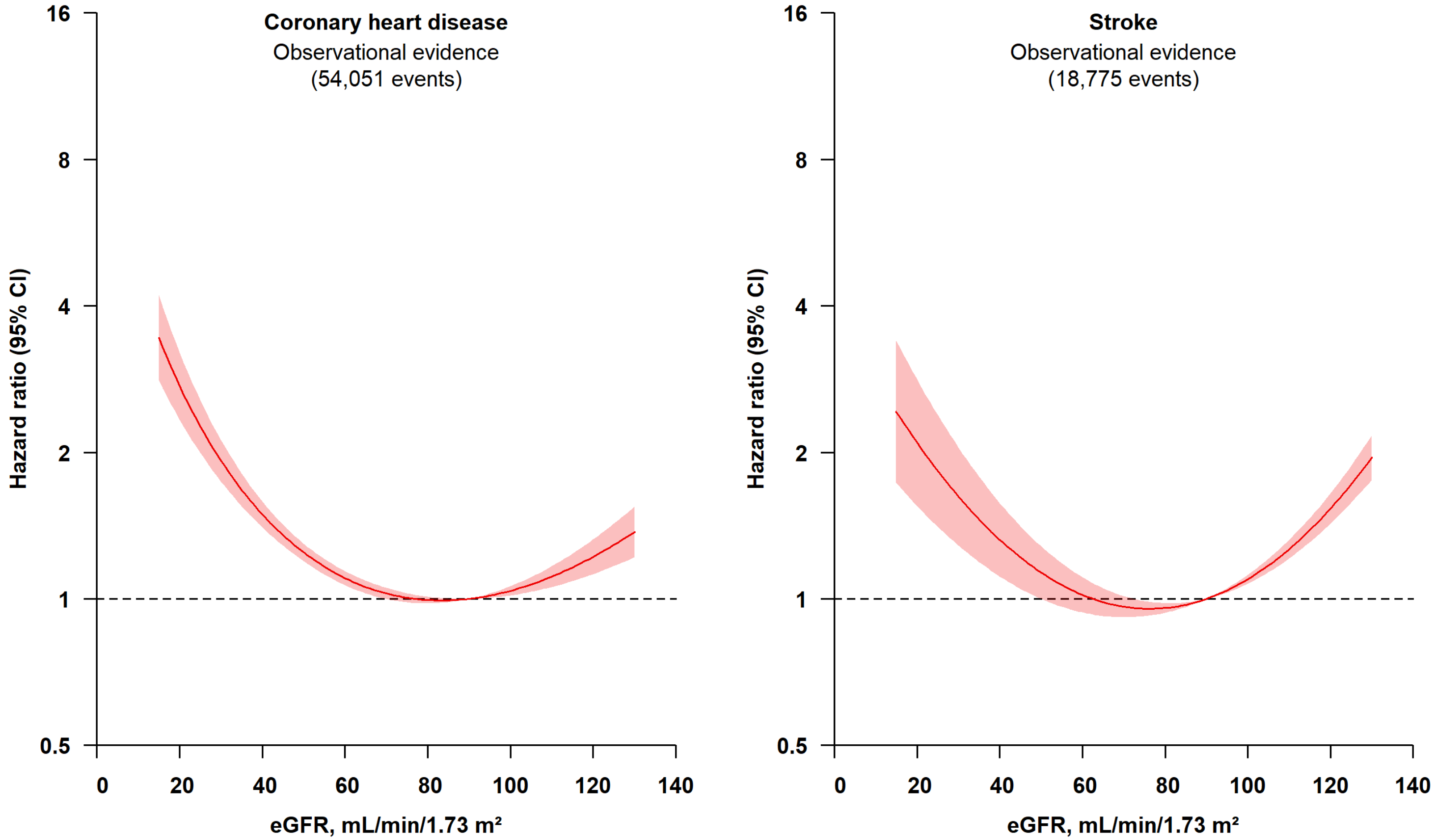

Participants with missing information on age, sex, systolic blood pressure, total and high-density lipoprotein cholesterol, body-mass index, smoking status were excluded from the analysis. Hazards ratios were estimated using Cox regression, adjusted for age, and systolic blood pressure, total and high-density lipoprotein cholesterol, body-mass index, and smoking status, and stratified by sex and study center. eGFR was estimated using creatinine-based CKD-EPI formula. The reference point is 90 mL/min/1.73 m^2. Shaded regions indicate 95% confidence interval.

Figure S7: Observational association of creatinine-based eGFR with risk of coronary heart disease and stroke, with / without complete information on vascular risk factors

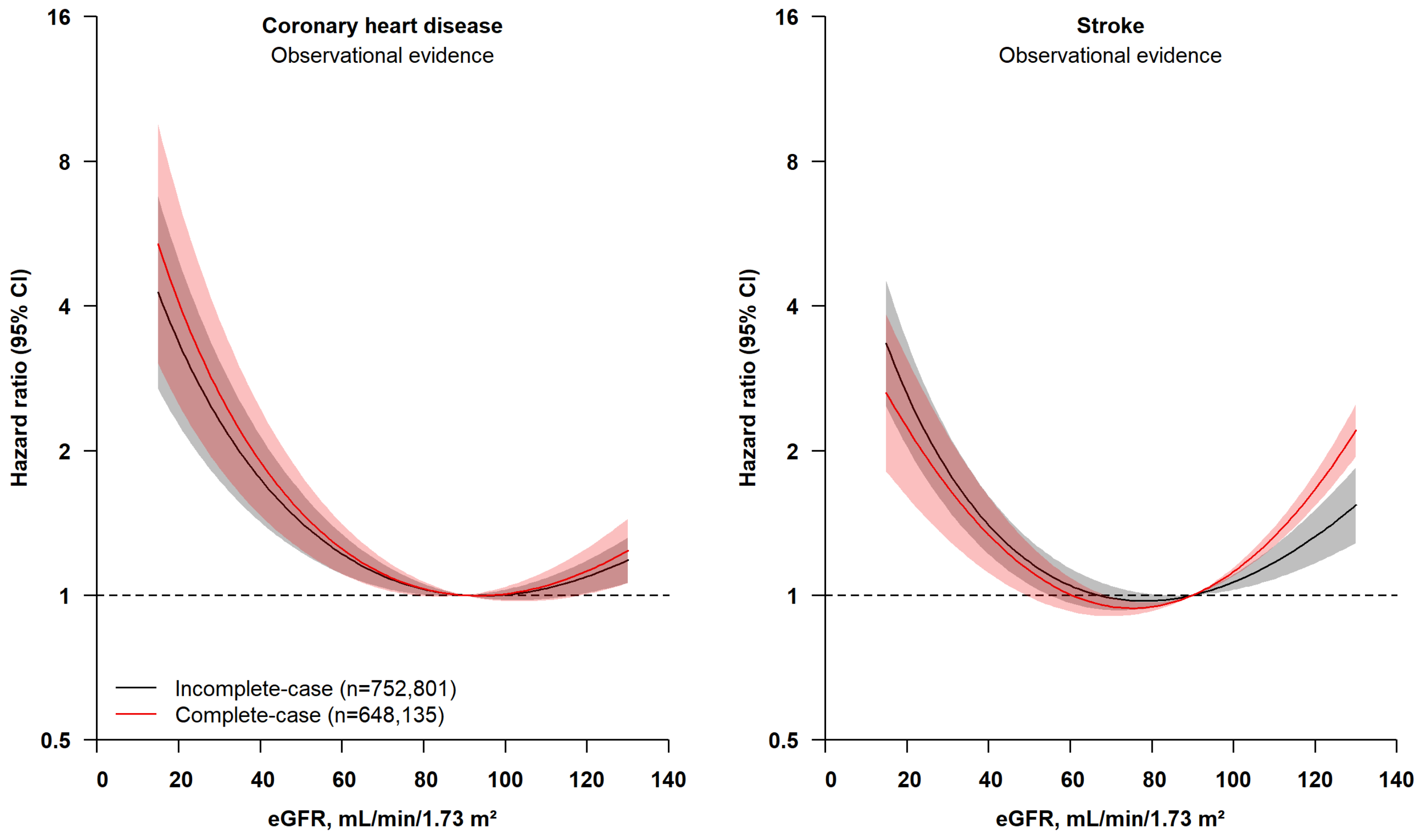

Incomplete-case included participants with information on age, sex, creatinine measurements; and complete-case included participants with complete information on age, sex, creatinine measurement, systolic blood pressure, total and high-density lipoprotein cholesterol, body-mass index, smoking status. Hazards ratios were estimated using Cox regression, adjusted for age, and systolic blood pressure, total and high-density lipoprotein cholesterol, body-mass index, and smoking status, and stratified by sex and study center. eGFR was estimated using creatinine-based CKD-EPI formula. The reference point is 90 mL/min/1.73 m^2. Shaded regions indicate 95% confidence intervals.

Figure S8: Observational associations of eGFR, estimated using creatinine or cystatin-C, with risk of coronary heart disease and stroke

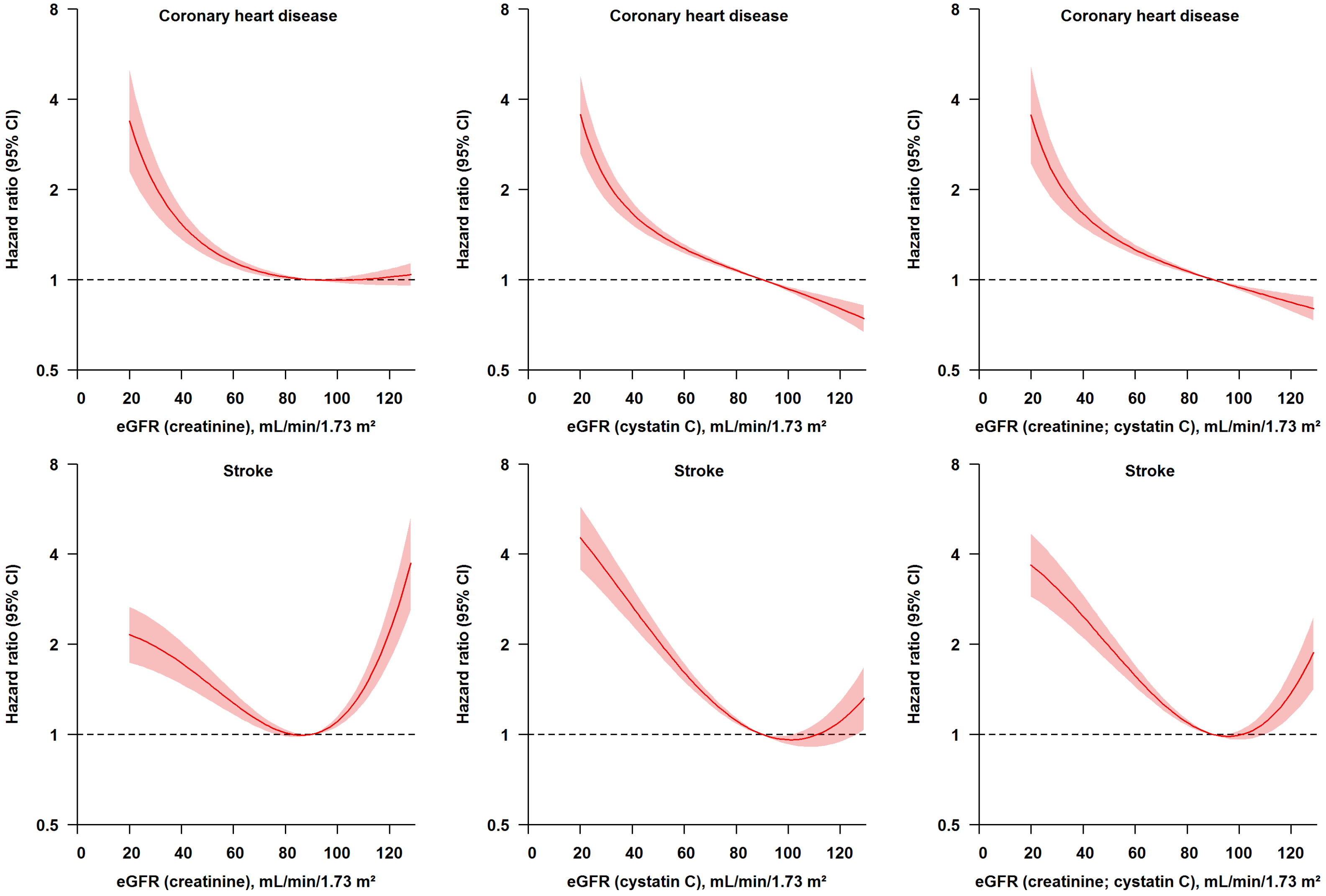

Participants with missing information on age, sex, systolic blood pressure, total and high-density lipoprotein cholesterol, body-mass index, smoking status were excluded from the analysis. Hazards ratios were estimated using Cox regression, adjusted for age, and systolic blood pressure, total and high-density lipoprotein cholesterol, body-mass index, and smoking status, and stratified by sex and study center. The reference point is 90 mL/min/1.73 m^2. Shaded regions indicate 95% confidence intervals.

Figure S9: Observational associations of urinary albumin and urinary albumin-creatinine ratio with risk of coronary heart disease and stroke in UK Biobank

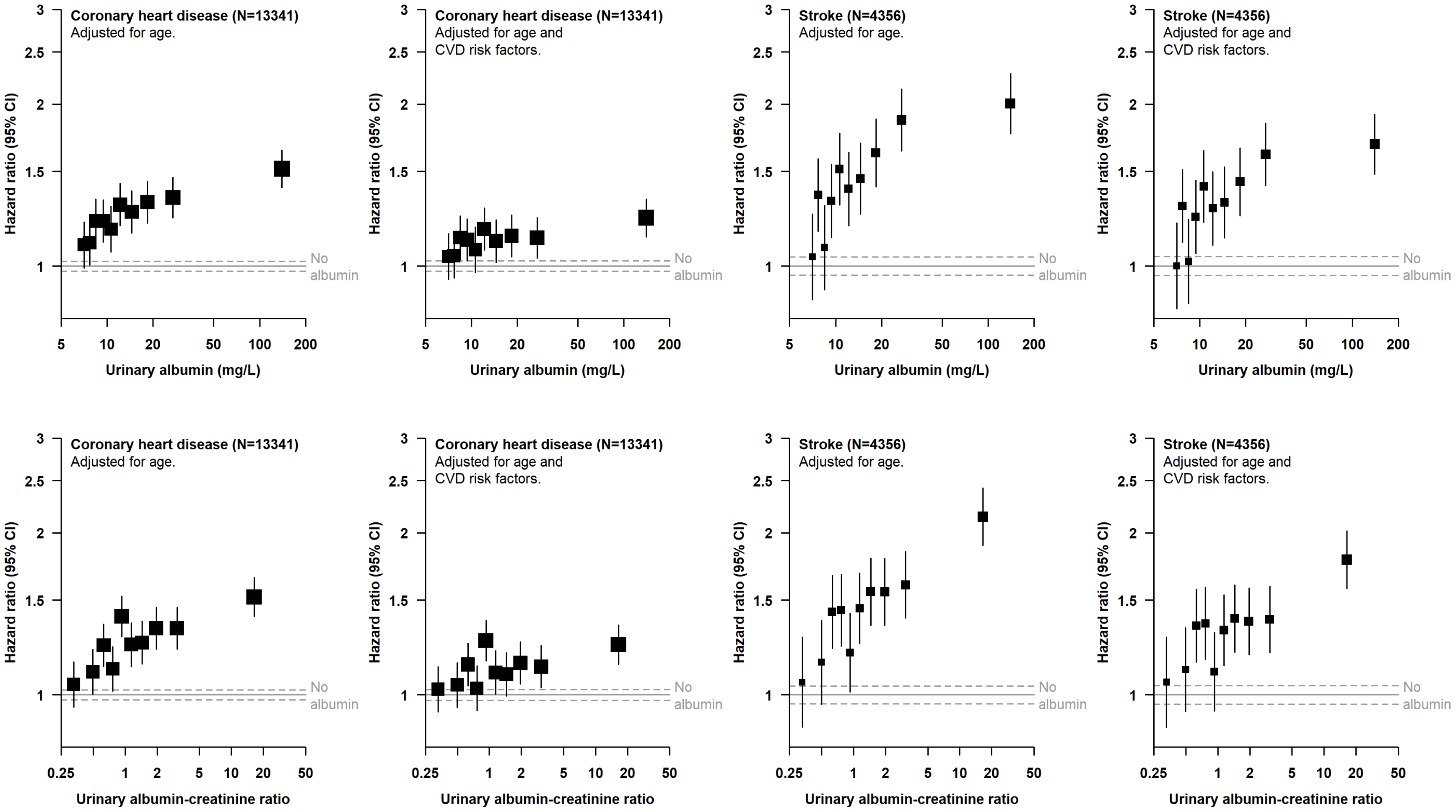

Participants with missing information on age, sex, systolic blood pressure, total and high-density lipoprotein cholesterol, body-mass index, smoking status were excluded from the analysis. Participants were divided into deciles based on their detectable urinary albumin value (i.e., ≥6.7 mmol/L), with an extra category grouping those with urinary albumin below the detection limit (indicated as no albumin). The group with no albumin measurements was the reference category. Hazards ratios were estimated using Cox regression, and plotted against the mean levels of the urinary marker in each category. Sizes of the boxes are proportional to the inverse of the variance of the log risk within that specific group. Vertical lines represent 95% confidence intervals. Dashed lines above and below 1 indicated the 95% confidence interval for the reference category calculated using the method of floating absolute risk.

Figure S10: Observational association of creatinine-based eGFR with risk of coronary heart disease and stroke in UK Biobank, by presence of urinary microalbumin

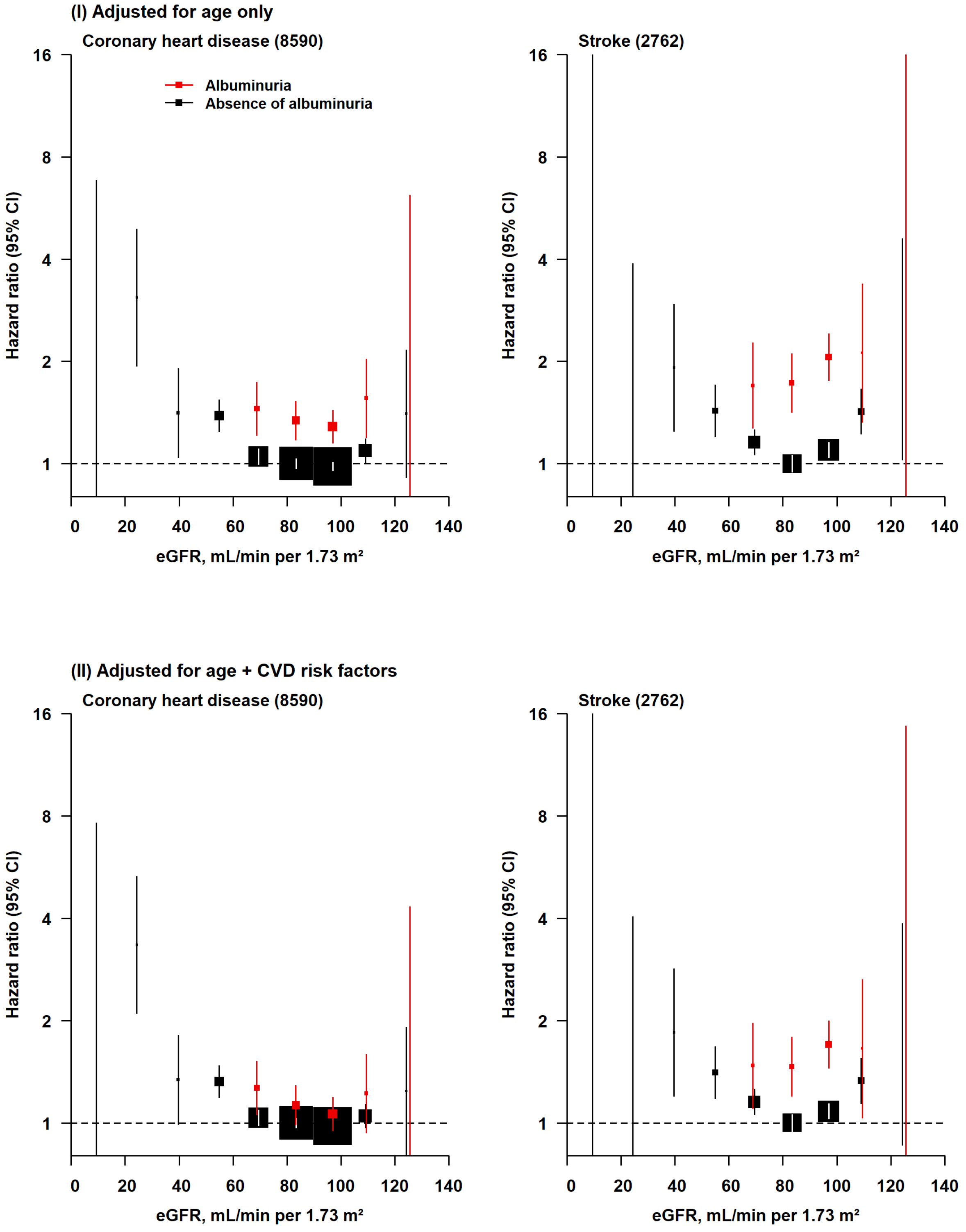

Participants with missing information on age, sex, systolic blood pressure, total and high-density lipoprotein cholesterol, body-mass index, smoking status were excluded from the analysis. Hazards ratios were estimated using Cox regression, adjusted for age, and stratified by sex. Full adjustment included further adjustment for systolic blood pressure, body-mass index, total cholesterol, HDL cholesterol, smoking, and use of lipid-lowering treatments. eGFR was estimated using creatinine-based CKD-EPI formula. The categories of eGFR are <15, 15-<30, 30-<45. 45-<60, 60-<75, 75-<90, 90-<105, 105-<120, and ≥120 mL/min/1.73 m^2. The reference category is 75-<90 mL/min.1.73 m^2 with no urinary microalbumin. Hazards ratios are plotted against the mean eGFR within that category, and vertical lines represent 95% confidence interval.

Figure S11: Associations of the genetic risk scores for eGFR with kidney function biomarkers and traditional vascular risk factors

		GRS for eGFR	GRS for eGFR (Cys)	GRS for eGFR (BUN)
**Kidney function biomarkers**	**No. of participants**	**Changes in SD (95% CI)**	**Changes in SD (95% CI)**	**Changes in SD (95% CI)**
creatinine-based eGFR	313,938	0.89 (0.87, 0.90)	0.74 (0.73, 0.76)	0.70 (0.69, 0.72)
Cystatin-C, mg/L	313,700	-0.59 (-0.61,-0.58)	-0.66 (-0.68,-0.65)	-0.58 (-0.60,-0.57)
Blood urea nitrogen, mmol/L	313,812	-0.34 (-0.36,-0.33)	-0.38 (-0.40,-0.36)	-0.46 (-0.47,-0.44)
Uric acid, umol/L	313,500	-0.24 (-0.25,-0.23)	-0.23 (-0.25,-0.22)	-0.26 (-0.28,-0.25)
**Traditional vascular risk factors**				
Waist-hip ratio	313,425	0.03 (0.02, 0.04)	0.03 (0.02, 0.05)	0.05 (0.04, 0.06)
Body-mass index, kg/m²	313,136	-0.02 (-0.03, 0.00)	-0.01 (-0.03, 0.01)	0.01 (-0.01, 0.03)
C-reactive protein, mg/L	313,213	0.01 (-0.01, 0.03)	-0.05 (-0.06,-0.03)	-0.03 (-0.04,-0.01)
Systolic blood pressure, mmHg	313,667	-0.03 (-0.04,-0.01)	-0.06 (-0.08,-0.04)	-0.05 (-0.06,-0.03)
Diastolic blood pressure, mmHg	313,668	-0.05 (-0.06,-0.03)	-0.08 (-0.10,-0.06)	-0.06 (-0.08,-0.04)
HDL-C, mmol/L	287,002	0.04 (0.02, 0.05)	0.03 (0.01, 0.04)	0.00 (-0.01, 0.02)
LDL-C, mmol/L	313,338	0.00 (-0.02, 0.02)	-0.01 (-0.02, 0.01)	-0.00 (-0.02, 0.01)
Triglycerides, mmol/L	313,813	-0.05 (-0.07,-0.03)	-0.03 (-0.05,-0.02)	-0.02 (-0.04,-0.00)
Lipoprotein (a), mg/dL	252,906	-0.11 (-0.13,-0.09)	-0.02 (-0.04, 0.00)	-0.01 (-0.03, 0.01)
HbA1c, %	299,917	-0.04 (-0.06,-0.03)	-0.06 (-0.08,-0.04)	-0.06 (-0.08,-0.05)
Diabetes	13,825 / 362,510	0.00 (-0.09, 0.09)	-0.02 (-0.11, 0.06)	0.01 (-0.07, 0.10)
Current-smoker	37,393 / 362,510	0.01 (-0.04, 0.06)	-0.03 (-0.09, 0.02)	-0.01 (-0.07, 0.04)

-1 0 1 Per 5-SD higher in GRS

GRS was constructed using 218 eGFR (creatinine-based) associated genetic variants reported in CKDGen (n=567,460). GRS (Cys) included 127 genetic variants (out of the 218 genetic variants included in the GRS for eGFR) that were additionally associated ($P<5x10^{-8}$) with cystatin-C-based eGFR (n=460,826). GRS (BUN) included 121 genetic variants (out of the 218 genetic variants included in the GRS for eGFR) that were additionally associated ($P<5x10^{-8}$) with blood urine nitrogen (n=416,178). The analyses were conducted in UKB and restricted to participants of European ancestry, not on lipid-lowering treatment, without prior history of cardiovascular diseases or diabetes at baseline, where appropriate. Analyses were adjusted for age, age-squared, sex, study centre, the first ten principal components. For continuous traits, general linear regression was used to estimate SD differences in all traits (after rank inverse normal transformation) per 1 SD. higher GRS. For binary traits, logistic regression was used to estimate log odds ratio differences per 1 SD. higher GRS. Results were shown for each per 5-SD higher in GRS. SD= standard derivation. GRS= genetic risk score.

Figure S12: Associations of the GRSs for eGFR with 167 NMR-measured metabolites

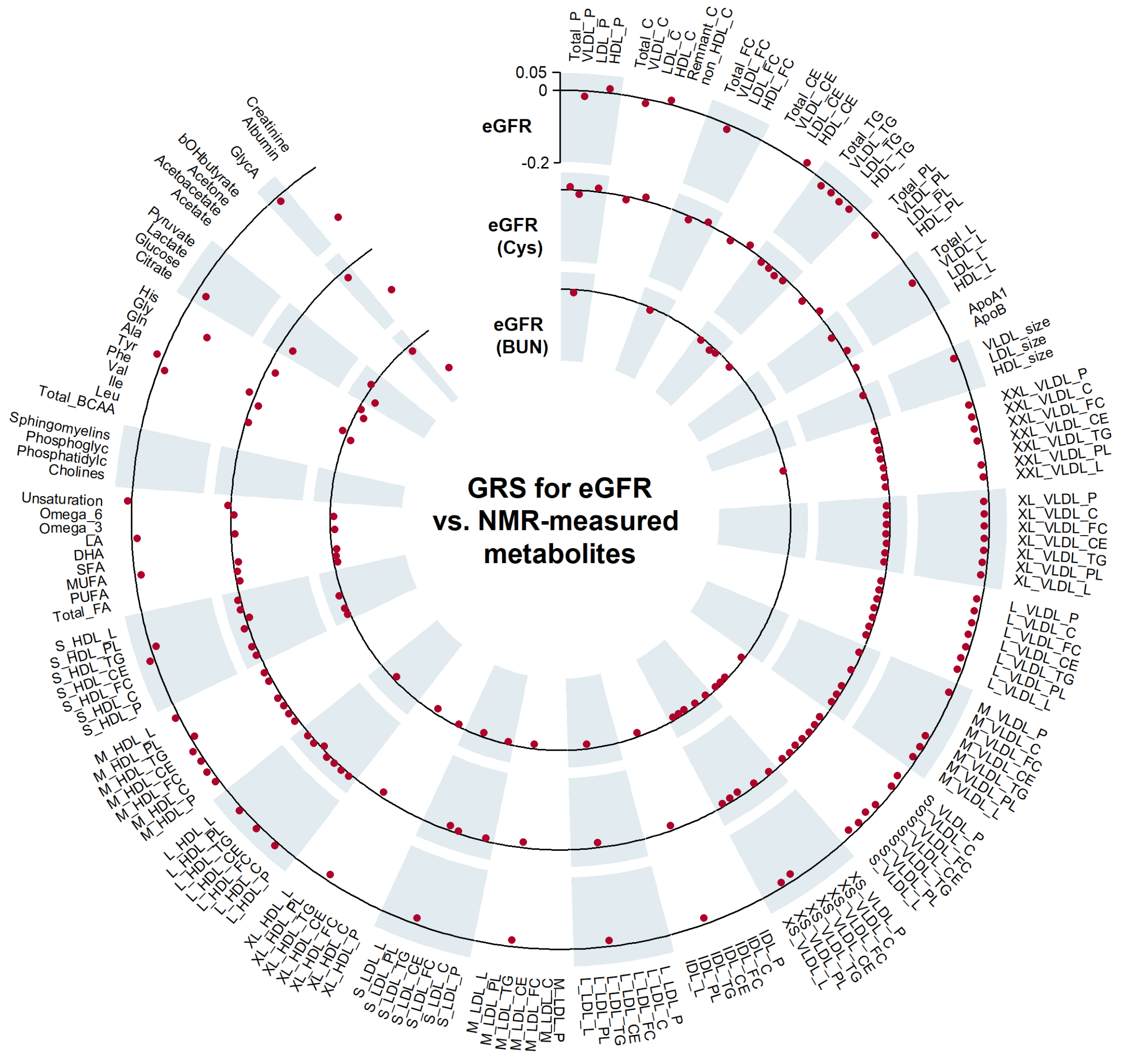

GRS was constructed using 218 eGFR (creatinine-based) associated genetic variants (n=567,460). GRS (Cys) included 127 genetic variants (out of the 218 genetic variants included in the GRS for eGFR) that were additionally associated with cystatin-C-based eGFR (n=460,826). GRS (BUN) included 121 genetic variants (out of the 218 genetic variants included in the GRS for eGFR) that were additionally associated with blood urine nitrogen (n=416,178). The analyses were conducted in a subset of UK Biobank study (n=79,413), with European ancestry, not on lipid-lowering treatments, and had no prior history of diabetes or vascular disease at baseline. Estimates were adjusted for age, age-squared, sex, study center, the first ten principal components. Points shown represent estimates for each metabolite per SD higher GRSs that fell below Bonferroni-corrected significant ($P<3.0x10^{-4}$). XXL= chylomicrons and extremely large. XL= extra large. L= large. M= medium. S= small. XS= very small. VLDL= very low-density lipoprotein. LDL= low-density lipoprotein. HDL= high-density lipoprotein. TG= triglycerides. P= particle concentrations. C= cholesterol. FC= free cholesterol. EC= esterified cholesterol. PL= phospholipids. L= total lipids. D= particle size. FA= fatty acids. LA= linoleic acid. PUFA= polyunsaturated fatty acids. MUFA= monounsaturated fatty acids.

Figure S13: Mendelian randomization estimates of genetically-predicted eGFR with risk of coronary heart disease and stroke (n=413,718)

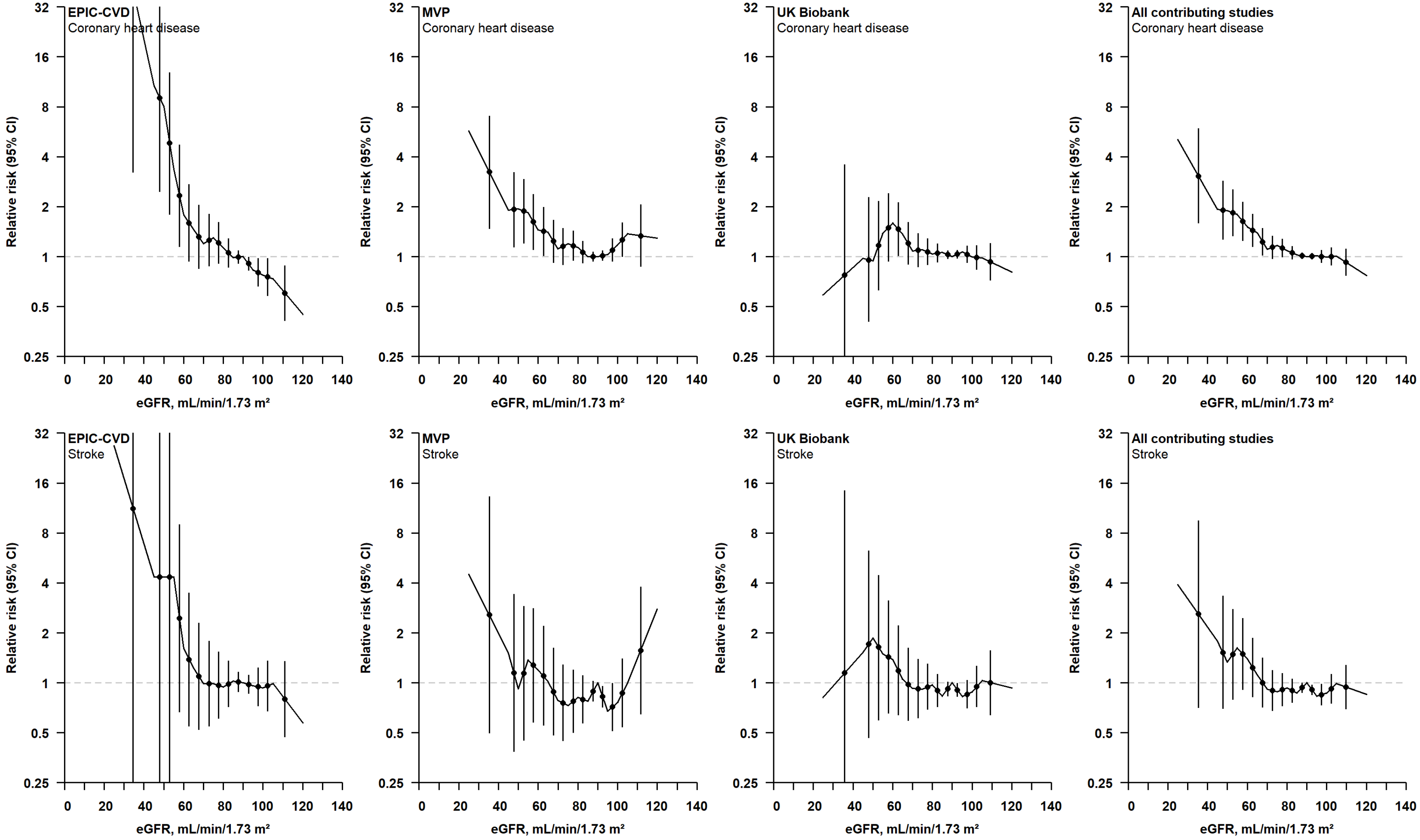

The reference point is 90 mL/min/1.73 m^2. Gradients at each point of the curve represent the localized average causal effect on coronary heart disease or stroke per 5 mL/min/1.73 m^2 change in genetically-predicted eGFR. Vertical lines represent 95% confidence intervals. Analyses were adjusted for age, age-squared, sex, study center, and the first ten principal components.

Figure S14: Comparison of non-linear Mendelian randomization methods.

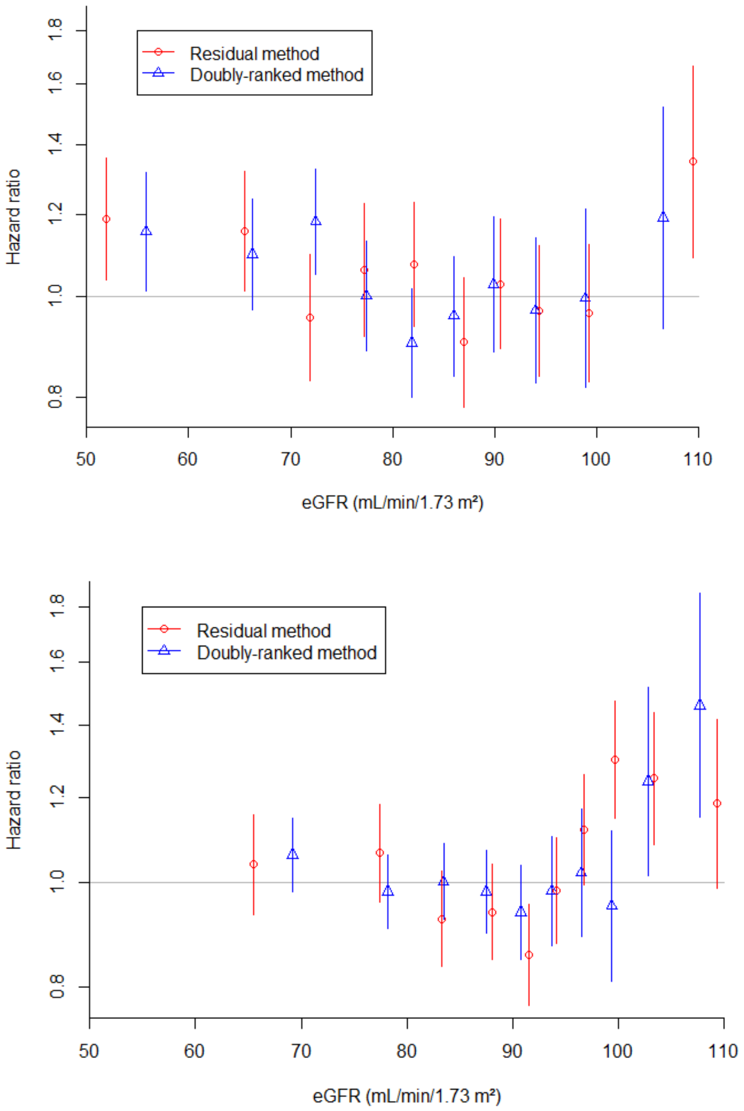

Hazard ratios from the residual method (red circles) and doubly-ranked method (blue triangles) representing the HR per 5 mL/min/1.73 m^2 lower genetically-predicted eGFR in decile groups: (top) Million Veteran Program, (bottom) UK Biobank. Error bars represent 95% confidence intervals.

Figure S15: Mendelian randomization estimates of genetically-predicted eGFR with risk of coronary heart disease, stroke, and ischemic stroke, *adjusted for other factors* (n=408,021)

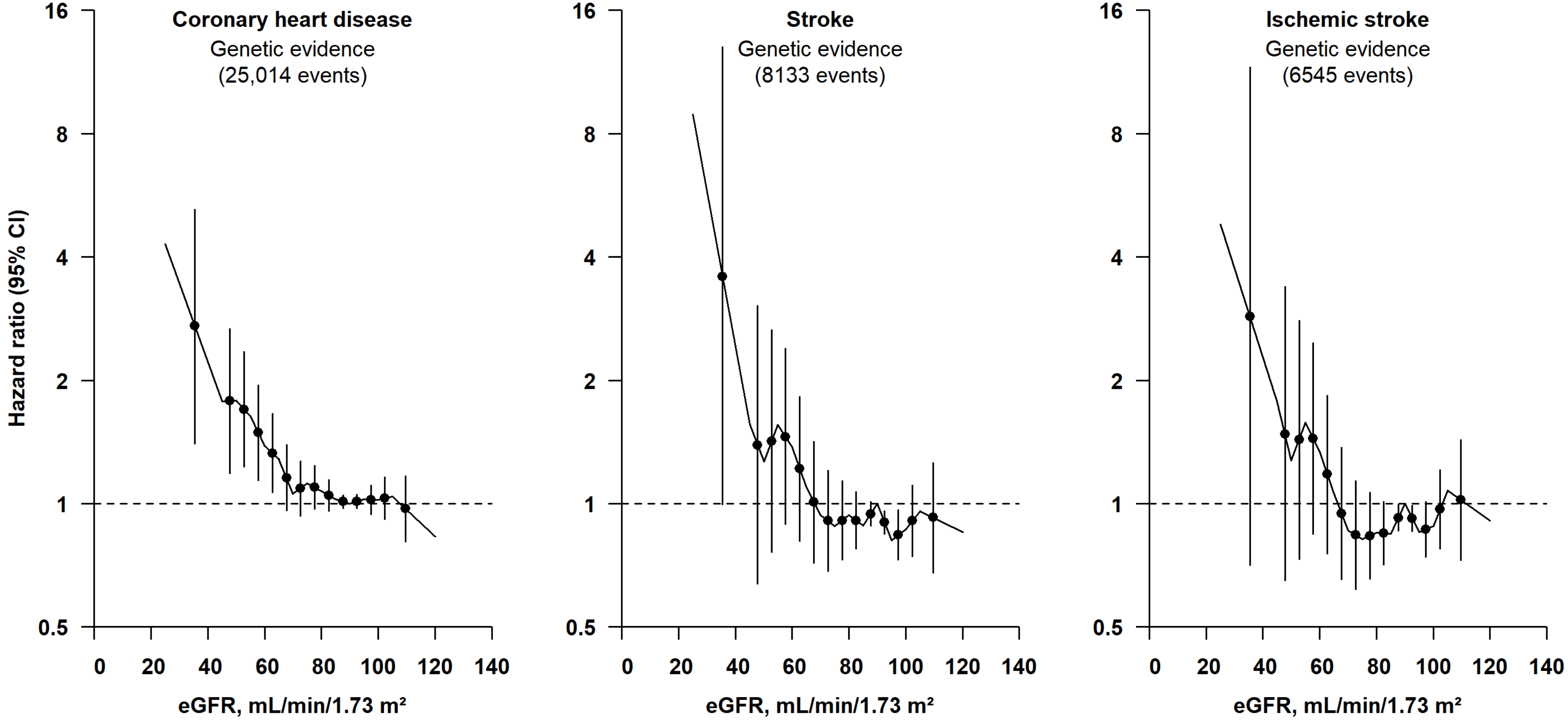

Stratum-specific localized average casual effect estimates were adjusted for age, age-squared, sex, study center, and the first ten principal components, with additional adjustment for vascular traits associated with the eGFR GRS, (systolic blood pressure, lipoprotein [a], hemoglobin A1c, and triglycerides). To maximize the number of participants with complete information on those vascular traits, we used genetically-predicted lipoprotein (a),[20] genetically-predicted hemoglobin A1c,[21] and genetically-predicted triglycerides,[22] instead of the measured levels. The reference point is 90 mL/min/1.73 m^2. Gradients at each point of the curve represent the localized average causal effect on coronary heart disease or stroke per 5 mL/min/1.73 m^2 change in genetically-predicted eGFR. Vertical lines represent 95% confidence intervals.

Figure S16: Mendelian randomization estimates of genetically-predicted eGFR with risk of coronary heart disease and stroke, *irrespective of diabetes status at recruitment* (n=463,051)

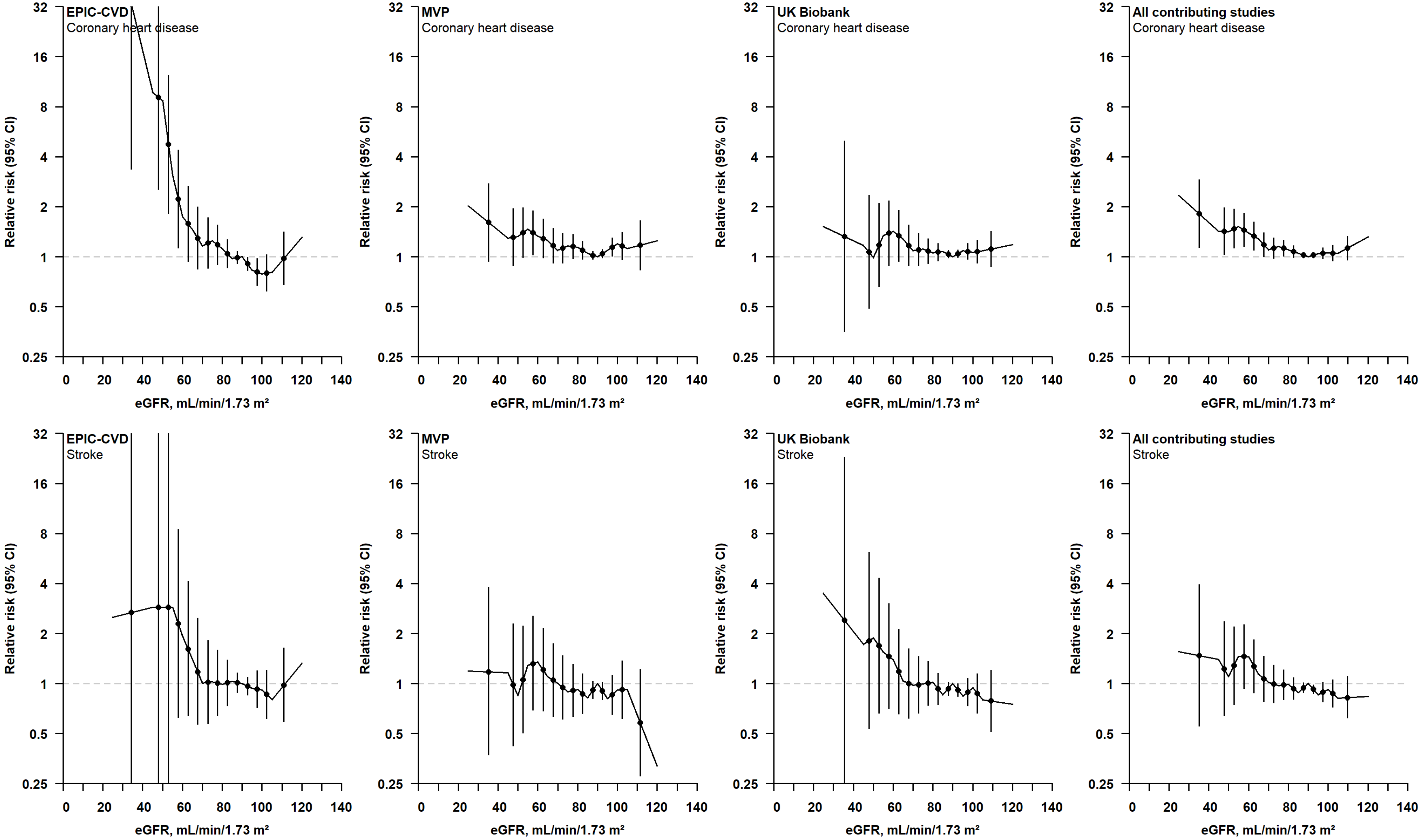

The reference point is 90 mL/min/1.73 m^2. Gradients at each point of the curve represent the localized average causal effect on coronary heart disease or stroke per 5 mL/min/1.73 m^2 change in genetically-predicted eGFR. Vertical lines represent 95% confidence intervals.

Figure S17: Mendelian randomization estimates of genetically-predicted eGFR with risk of coronary heart disease, stroke, and ischemic stroke (n=413,718)

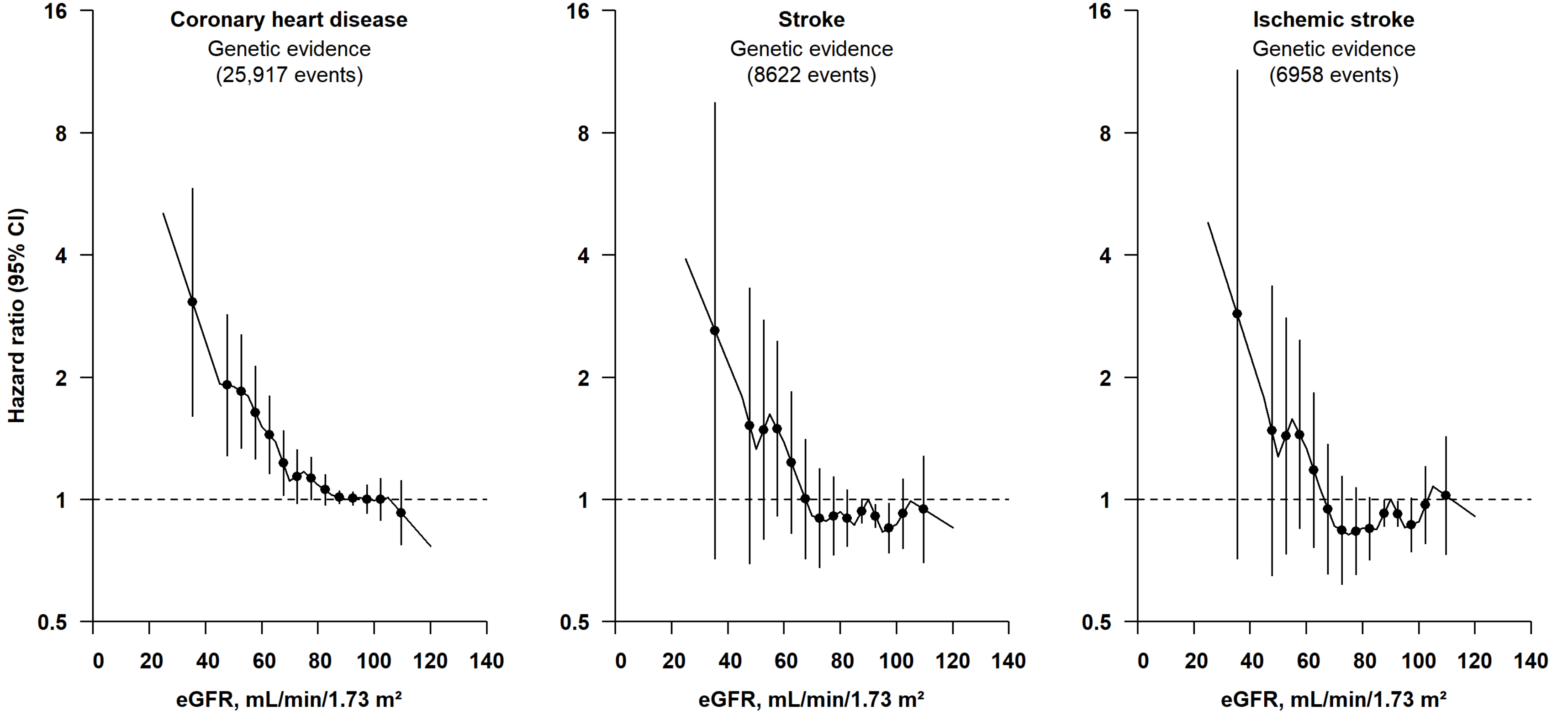

The reference point is 90 mL/min/1.73 m^2. Gradients at each point of the curve represent the localized average causal effect on coronary heart disease or stroke per 5 mL/min/1.73 m^2 change in genetically-predicted eGFR. Vertical lines represent 95% confidence intervals.

Figure S18: Mendelian randomization estimates of genetically-predicted eGFR with risk of coronary heart disease, stroke, and ischemic stroke, by different GRSs

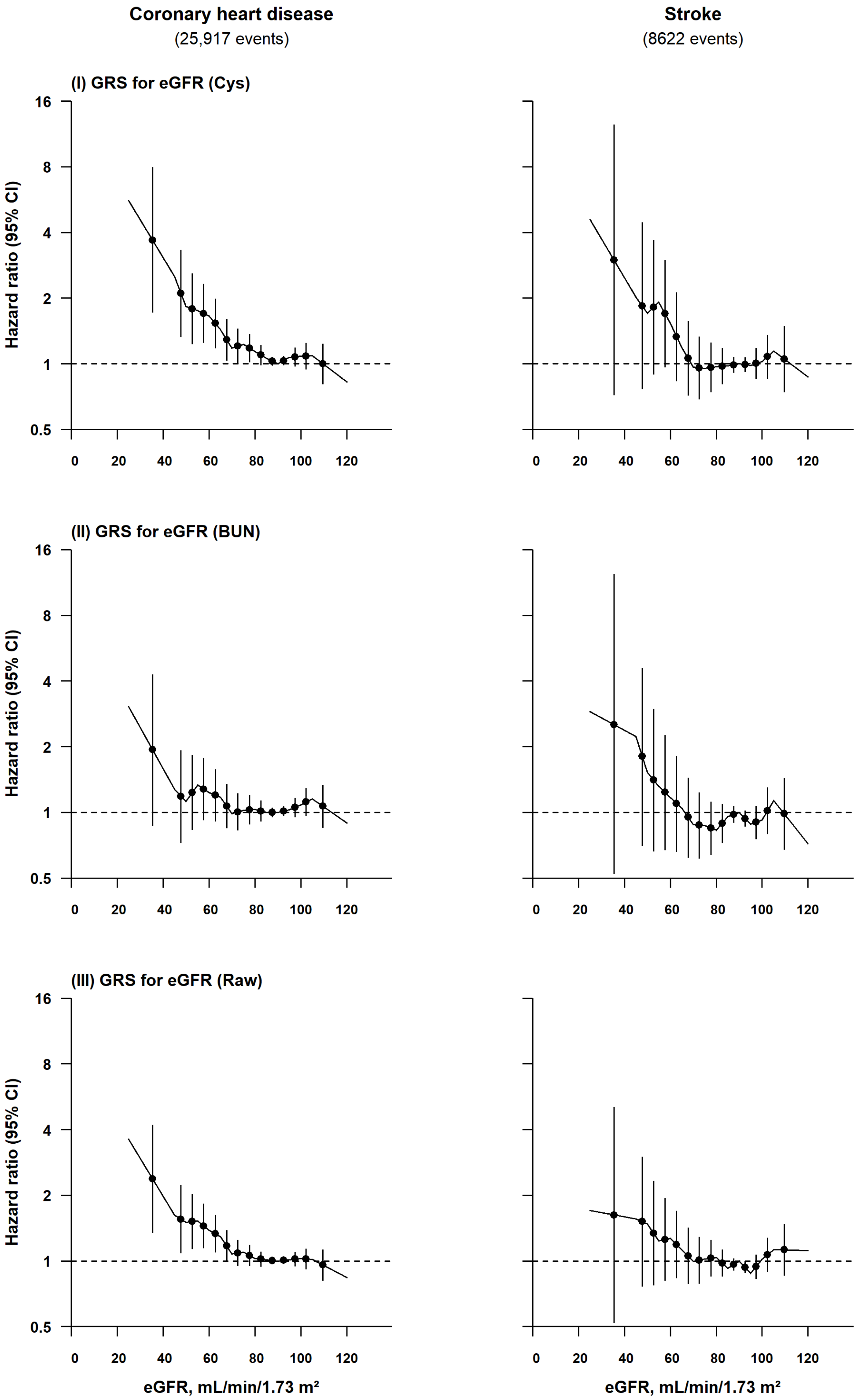

The reference point is 90 mL/min/1.73 m^2. Gradients at each point of the curve represent the localized average causal effect on coronary heart disease or stroke per 5 mL/min/1.73 m^2 changes in genetically-predicted eGFR. Vertical lines represent 95% confidence intervals.

ACKNOWLEDGEMENTS

VA Million Veteran Program
Core Acknowledgement for Publications
Updated December 2021

MVP Program Office
- Program Director - Sumitra Muralidhar, Ph.D.
  US Department of Veterans Affairs, 810 Vermont Avenue NW, Washington, DC 20420
- Associate Director, Scientific Programs - Jennifer Moser, Ph.D.
  US Department of Veterans Affairs, 810 Vermont Avenue NW, Washington, DC 20420
- Associate Director, Cohort Management & Public Relations - Jennifer E. Deen, B.S.
  US Department of Veterans Affairs, 810 Vermont Avenue NW, Washington, DC 20420

MVP Executive Committee
- Co-Chair: J. Michael Gaziano, M.D., M.P.H.
  VA Boston Healthcare System, 150 S. Huntington Avenue, Boston, MA 02130
- Co-Chair: Sumitra Muralidhar, Ph.D.
  US Department of Veterans Affairs, 810 Vermont Avenue NW, Washington, DC 20420
- Jean Beckham, Ph.D.
  Durham VA Medical Center, 508 Fulton Street, Durham, NC 27705
- Kyong-Mi Chang, M.D.
  Philadelphia VA Medical Center, 3900 Woodland Avenue, Philadelphia, PA 19104
- Philip S. Tsao, Ph.D.
  VA Palo Alto Health Care System, 3801 Miranda Avenue, Palo Alto, CA 94304
- Shiuh-Wen Luoh, M.D., Ph.D.
  VA Portland Health Care System, 3710 SW US Veterans Hospital Rd, Portland, OR 97239
  US Department of Veterans Affairs, 810 Vermont Avenue NW, Washington, DC 20420
- Juan P. Casas, M.D., Ph.D., Ex-Officio
  VA Boston Healthcare System, 150 S. Huntington Avenue, Boston, MA 02130

MVP Principal Investigators
- J. Michael Gaziano, M.D., M.P.H.
  VA Boston Healthcare System, 150 S. Huntington Avenue, Boston, MA 02130
- Philip S. Tsao, Ph.D.
  VA Palo Alto Health Care System, 3801 Miranda Avenue, Palo Alto, CA 94304

MVP Operations
- MVP Executive Director – Juan P. Casas, M.D., Ph.D.
  VA Boston Healthcare System, 150 S. Huntington Avenue, Boston, MA 02130
- Director of Regulatory Affairs – Lori Churby, B.S.
  VA Palo Alto Health Care System, 3801 Miranda Avenue, Palo Alto, CA 94304
- MVP Cohort Management Director – Stacey B. Whitbourne, Ph.D.

VA Boston Healthcare System, 150 S. Huntington Avenue, Boston, MA 02130
- MVP Recruitment/Enrollment Director - Jessica V. Brewer, M.P.H.
  VA Boston Healthcare System, 150 S. Huntington Avenue, Boston, MA 02130
- Director, VA Central Biorepository, Boston – Mary T. Brophy M.D., M.P.H.
  VA Boston Healthcare System, 150 S. Huntington Avenue, Boston, MA 02130
- Executive Director for MVP Biorepositories - Luis E. Selva, Ph.D.
  VA Boston Healthcare System, 150 S. Huntington Avenue, Boston, MA 02130
- MVP Informatics, Boston – Shahpoor (Alex) Shayan, M.S.
  VA Boston Healthcare System, 150 S. Huntington Avenue, Boston, MA 02130
- Director, MVP Data Operations/Analytics, Boston – Kelly Cho, M.P.H., Ph.D.
  VA Boston Healthcare System, 150 S. Huntington Avenue, Boston, MA 02130
- Director, Center for Computational and Data Science (C-DACS) & Genomics Core – Saiju Pyarajan Ph.D.
  VA Boston Healthcare System, 150 S. Huntington Avenue, Boston, MA 02130
- Director, Molecular Data Core – Philip S. Tsao, Ph.D.
  VA Palo Alto Health Care System, 3801 Miranda Avenue, Palo Alto, CA 94304
- Director, Phenomics Data Core – Kelly Cho, M.P.H, Ph.D.
  VA Boston Healthcare System, 150 S. Huntington Avenue, Boston, MA 02130
- Director, VA Informatics and Computing Infrastructure (VINCI) – Scott L. DuVall, Ph.D.
  VA Salt Lake City Health Care System, 500 Foothill Drive, Salt Lake City, UT 84148
- MVP Coordinating Centers
  - Cooperative Studies Program Clinical Research Pharmacy Coordinating Center, Albuquerque – Todd Connor, Pharm.D.; Dean P. Argyres, B.S., M.S.
    New Mexico VA Health Care System, 1501 San Pedro Drive SE, Albuquerque, NM 87108
  - Genomics Coordinating Center, Palo Alto – Philip S. Tsao, Ph.D.
    VA Palo Alto Health Care System, 3801 Miranda Avenue, Palo Alto, CA 94304
  - MVP Boston Coordinating Center, Boston - J. Michael Gaziano, M.D., M.P.H.
    VA Boston Healthcare System, 150 S. Huntington Avenue, Boston, MA 02130
  - MVP Information Center, Canandaigua – Brady Stephens, M.S.
    Canandaigua VA Medical Center, 400 Fort Hill Avenue, Canandaigua, NY 14424

Current MVP Local Site Investigators
- Atlanta VA Medical Center (Peter Wilson, M.D.)
  1670 Clairmont Road, Decatur, GA 30033
- Bay Pines VA Healthcare System (Rachel McArdle, Ph.D.)
  10,000 Bay Pines Blvd Bay Pines, FL 33744
- Birmingham VA Medical Center (Louis Dellitalia, M.D.)
  700 S. 19th Street, Birmingham AL 35233
- Central Western Massachusetts Healthcare System (Kristin Mattocks, Ph.D., M.P.H.)
  421 North Main Street, Leeds, MA 01053
- Cincinnati VA Medical Center (John Harley, M.D., Ph.D.)

3200 Vine Street, Cincinnati, OH 45220

- Clement J. Zablocki VA Medical Center (Jeffrey Whittle, M.D., M.P.H.)
  5000 West National Avenue, Milwaukee, WI 53295
- VA Northeast Ohio Healthcare System (Frank Jacono, M.D.)
  10701 East Boulevard, Cleveland, OH 44106
- Durham VA Medical Center (Jean Beckham, Ph.D.)
  508 Fulton Street, Durham, NC 27705
- Edith Nourse Rogers Memorial Veterans Hospital (John Wells., Ph.D.)
  200 Springs Road, Bedford, MA 01730
- Edward Hines, Jr. VA Medical Center (Salvador Gutierrez, M.D.)
  5000 South 5th Avenue, Hines, IL 60141
- Veterans Health Care System of the Ozarks (Kathrina Alexander, M.D.)
  1100 North College Avenue, Fayetteville, AR 72703
- Fargo VA Health Care System (Kimberly Hammer, Ph.D.)
  2101 N. Elm, Fargo, ND 58102
- VA Health Care Upstate New York (James Norton, Ph.D.)
  113 Holland Avenue, Albany, NY 12208
- New Mexico VA Health Care System (Gerardo Villareal, M.D.)
  1501 San Pedro Drive, S.E. Albuquerque, NM 87108
- VA Boston Healthcare System (Scott Kinlay, M.B.B.S., Ph.D.)
  150 S. Huntington Avenue, Boston, MA 02130
- VA Western New York Healthcare System (Junzhe Xu, M.D.)
  3495 Bailey Avenue, Buffalo, NY 14215-1199
- Ralph H. Johnson VA Medical Center (Mark Hamner, M.D.)
  109 Bee Street, Mental Health Research, Charleston, SC 29401
- Columbia VA Health Care System (Roy Mathew, M.D.)
  6439 Garners Ferry Road, Columbia, SC 29209
- VA North Texas Health Care System (Sujata Bhushan, M.D.)
  4500 S. Lancaster Road, Dallas, TX 75216
- Hampton VA Medical Center (Pran Iruvanti, D.O., Ph.D.)
  100 Emancipation Drive, Hampton, VA 23667
- Richmond VA Medical Center (Michael Godschalk, M.D.)
  1201 Broad Rock Blvd., Richmond, VA 23249
- Iowa City VA Health Care System (Zuhair Ballas, M.D.)
  601 Highway 6 West, Iowa City, IA 52246-2208
- Eastern Oklahoma VA Health Care System (River Smith, Ph.D.)
  1011 Honor Heights Drive, Muskogee, OK 74401
- James A. Haley Veterans’ Hospital (Stephen Mastorides, M.D.)
  13000 Bruce B. Downs Blvd, Tampa, FL 33612
- James H. Quillen VA Medical Center (Jonathan Moorman, M.D., Ph.D.)
  Corner of Lamont & Veterans Way, Mountain Home, TN 37684
- John D. Dingell VA Medical Center (Saib Gappy, M.D.)
  4646 John R Street, Detroit, MI 48201

- Louisville VA Medical Center (Jon Klein, M.D., Ph.D.)
  800 Zorn Avenue, Louisville, KY 40206
- Manchester VA Medical Center (Nora Ratcliffe, M.D.)
  718 Smyth Road, Manchester, NH 03104
- Miami VA Health Care System (Ana Palacio, M.D., M.P.H.)
  1201 NW 16th Street, 11 GRC, Miami FL 33125
- Michael E. DeBakey VA Medical Center (Olaoluwa Okusaga, M.D.)
  2002 Holcombe Blvd, Houston, TX 77030
- Minneapolis VA Health Care System (Maureen Murdoch, M.D., M.P.H.)
  One Veterans Drive, Minneapolis, MN 55417
- N. FL/S. GA Veterans Health System (Peruvemba Sriram, M.D.)
  1601 SW Archer Road, Gainesville, FL 32608
- Northport VA Medical Center (Shing Shing Yeh, Ph.D., M.D.)
  79 Middleville Road, Northport, NY 11768
- Overton Brooks VA Medical Center (Neeraj Tandon, M.D.)
  510 East Stoner Ave, Shreveport, LA 71101
- Philadelphia VA Medical Center (Darshana Jhala, M.D.)
  3900 Woodland Avenue, Philadelphia, PA 19104
- Phoenix VA Health Care System (Samuel Aguayo, M.D.)
  650 E. Indian School Road, Phoenix, AZ 85012
- Portland VA Medical Center (David Cohen, M.D.)
  3710 SW U.S. Veterans Hospital Road, Portland, OR 97239
- Providence VA Medical Center (Satish Sharma, M.D.)
  830 Chalkstone Avenue, Providence, RI 02908
- Richard Roudebush VA Medical Center (Suthat Liangpunsakul, M.D., M.P.H.)
  1481 West 10th Street, Indianapolis, IN 46202
- Salem VA Medical Center (Kris Ann Oursler, M.D.)
  1970 Roanoke Blvd, Salem, VA 24153
- San Francisco VA Health Care System (Mary Whooley, M.D.)
  4150 Clement Street, San Francisco, CA 94121
- South Texas Veterans Health Care System (Sunil Ahuja, M.D.)
  7400 Merton Minter Boulevard, San Antonio, TX 78229
- Southeast Louisiana Veterans Health Care System (Joseph Constans, Ph.D.)
  2400 Canal Street, New Orleans, LA 70119
- Southern Arizona VA Health Care System (Paul Meyer, M.D., Ph.D.)
  3601 S 6th Avenue, Tucson, AZ 85723
- Sioux Falls VA Health Care System (Jennifer Greco, M.D.)
  2501 W 22nd Street, Sioux Falls, SD 57105
- St. Louis VA Health Care System (Michael Rauchman, M.D.)
  915 North Grand Blvd, St. Louis, MO 63106
- Syracuse VA Medical Center (Richard Servatius, Ph.D.)
  800 Irving Avenue, Syracuse, NY 13210
- VA Eastern Kansas Health Care System (Melinda Gaddy, Ph.D.)

4101 S 4th Street Trafficway, Leavenworth, KS 66048

- VA Greater Los Angeles Health Care System (Agnes Wallbom, M.D., M.S.)
  11301 Wilshire Blvd, Los Angeles, CA 90073
- VA Long Beach Healthcare System (Timothy Morgan, M.D.)
  5901 East 7th Street Long Beach, CA 90822
- VA Maine Healthcare System (Todd Stapley, D.O.)
  1 VA Center, Augusta, ME 04330
- VA New York Harbor Healthcare System (Peter Liang, M.D., M.P.H.)
  423 East 23rd Street, New York, NY 10010
- VA Pacific Islands Health Care System (Daryl Fujii, Ph.D.)
  459 Patterson Rd, Honolulu, HI 96819
- VA Palo Alto Health Care System (Philip Tsao, Ph.D.)
  3801 Miranda Avenue, Palo Alto, CA 94304-1290
- VA Pittsburgh Health Care System (Patrick Strollo, Jr., M.D.)
  University Drive, Pittsburgh, PA 15240
- VA Puget Sound Health Care System (Edward Boyko, M.D.)
  1660 S. Columbian Way, Seattle, WA 98108-1597
- VA Salt Lake City Health Care System (Jessica Walsh, M.D.)
  500 Foothill Drive, Salt Lake City, UT 84148
- VA San Diego Healthcare System (Samir Gupta, M.D., M.S.C.S.)
  3350 La Jolla Village Drive, San Diego, CA 92161
- VA Sierra Nevada Health Care System (Mostaqul Huq, Pharm.D., Ph.D.)
  975 Kirman Avenue, Reno, NV 89502
- VA Southern Nevada Healthcare System (Joseph Fayad, M.D.)
  6900 North Pecos Road, North Las Vegas, NV 89086
- VA Tennessee Valley Healthcare System (Adriana Hung, M.D., M.P.H.)
  1310 24th Avenue, South Nashville, TN 37212
- Washington DC VA Medical Center (Jack Lichy, M.D., Ph.D.)
  50 Irving St, Washington, D. C. 20422
- W.G. (Bill) Hefner VA Medical Center (Robin Hurley, M.D.)
  1601 Brenner Ave, Salisbury, NC 28144
- White River Junction VA Medical Center (Brooks Robey, M.D.)
  163 Veterans Drive, White River Junction, VT 05009
- William S. Middleton Memorial Veterans Hospital (Prakash Balasubramanian, M.D.)
  2500 Overlook Terrace, Madison, WI 53705
